# Predicting fine-grained cell types from histology images through cross-modal learning in spatial transcriptomics

**DOI:** 10.1093/bioinformatics/btaf201

**Published:** 2025-07-15

**Authors:** Chaoyang Yan, Zhihan Ruan, Songkang Chen, Yichen Pan, Xue Han, Yuanyu Li, Jian Liu

**Affiliations:** College of Computer Science, Nankai University, Tianjin 300350, China; Centre for Bioinformatics and Intelligent Medicine, Nankai University, Tianjin 300350, China; College of Computer Science, Nankai University, Tianjin 300350, China; Centre for Bioinformatics and Intelligent Medicine, Nankai University, Tianjin 300350, China; College of Computer Science, Nankai University, Tianjin 300350, China; Centre for Bioinformatics and Intelligent Medicine, Nankai University, Tianjin 300350, China; College of Computer Science, Nankai University, Tianjin 300350, China; Centre for Bioinformatics and Intelligent Medicine, Nankai University, Tianjin 300350, China; College of Computer Science, Nankai University, Tianjin 300350, China; Centre for Bioinformatics and Intelligent Medicine, Nankai University, Tianjin 300350, China; College of Computer Science, Nankai University, Tianjin 300350, China; Centre for Bioinformatics and Intelligent Medicine, Nankai University, Tianjin 300350, China; State Key Laboratory of Medicinal Chemical Biology, College of Computer Science, Nankai University, Tianjin 300350, China

## Abstract

**Motivation:**

Fine-grained cellular characterization provides critical insights into biological processes, including tissue development, disease progression, and treatment responses. The spatial organization of cells and the interactions among distinct cell types play a pivotal role in shaping the tumor micro-environment, driving heterogeneity, and influencing patient prognosis. While computational pathology can uncover morphological structures from tissue images, conventional methods are often restricted to identifying coarse-grained and limited cell types. In contrast, spatial transcriptomics-based approaches hold promise for pinpointing fine-grained transcriptional cell types using histology data. However, these methods tend to overlook key molecular signatures inherent in gene expression data.

**Results:**

To this end, we propose a cross-modal unified representation learning framework (CUCA) for identifying fine-grained cell types from histology images. CUCA is trained on paired morphology-molecule spatial transcriptomics data, enabling it to infer fine-grained cell types solely from pathology images. Our model aims to harness the cross-modal embedding alignment paradigm to harmonize the embedding spaces of morphological and molecular modalities, bridging the gap between image patterns and molecular expression signatures. Extensive results across three datasets show that CUCA captures molecule-enhanced cross-modal representations and improves the prediction of fine-grained transcriptional cell abundances. Downstream analyses of cellular spatial architectures and intercellular co-localization reveal that CUCA provides insights into tumor biology, offering potential advancements in cancer research.

**Availability and implementation:**

The source code of CUCA is available in Zenodo: 10.5281/zenodo.15087256.

## 1 Introduction

Cells are the fundamental units of life, forming the structural and functional basis of tissues and organs in living organisms. The cellular architecture of tissues, which refers to the spatial arrangement and morphological characteristics of cells, offers critical insights into how cellular interactions contribute to biological behaviors, including tissue development, disease progression, and treatment responses (Rao *et al.* 2021, Gulati *et al.* 2025). In cancer research, cellular spatial architecture can elucidate key features of the tumor microenvironment (TME), heterogeneity, tumor lymphocyte infiltration, and their implications for patient prognosis and personalized treatment strategies (Zhao *et al.* 2023). Therefore, the accurate identification of cell types is essential for advancing our understanding of complex biological mechanisms and improving cancer management.

Current methods for cell type identification can be broadly categorized into two groups. (i) Computational pathology-based approaches that achieve nuclear instance segmentation and classification via deep learning (Graham *et al.* 2019, Hörst *et al.* 2024). While these methods can identify cell types in tissue images, they are limited to recognizing coarse-grained categories, typically no more than five major cell types. This restricts exploration of finer cellular subtypes. Furthermore, the position annotations for cell types are manually outlined, which compromises the reliability of the results. (ii) Approaches based on Spatial Transcriptomics (ST), such as Cell2location (Kleshchevnikov *et al.* 2022), Tangram (Biancalani *et al.* 2021), and RCTD (Cable *et al.* 2022), typically use cellular deconvolution algorithms (Li *et al.* 2023) to estimate the proportion of cell types within each ST spot using gene expression data. These methods integrate ST data with single-cell RNA sequencing (scRNA-seq) reference transcriptome signatures (Ruan *et al.* 2024a) and resolve fine-grained cell types based on transcriptomic profiles. However, existing methods primarily rely on gene expression and fail to exploit the rich morphological patterns present in histology images. Moreover, mainstream sequencing-based ST data suffer from a limitation of low spatial resolution.

Recent advancements have focused on combining paired histology images with molecular expression data to enhance spatial resolution for ST analysis. Bergenstråhle *et al.* (2022) presented a deep generative model to infer higher-resolution expression maps by integrating ST data with histological image data. They further enhanced spatial gene expression to near-single-cell resolution through a hierarchical vision transformer (Zhang *et al.* 2024). Similarly, Wan *et al.* (2023) proposed a deep generative model designed to attain single-cell resolution in ST enhancement. These studies have demonstrated that histological image features are correlated with molecular expression profiles, enabling the prediction of gene expression directly from histology images. This is promising as histopathological whole slide images (WSIs) are cost-effective and routinely used in clinical practice. Deep learning algorithms that incorporate both local and global morphological features, along with spatial relationships, have been developed to predict local gene expression directly from histopathology images (Jia *et al.* 2023). Although these methods achieved gene expression prediction and enabled downstream analyses, such as spatial domain identification and cell proportion estimation, the number of genes with predictive capabilities remains limited (Jaume *et al.* 2024). This curtails the comprehensive estimation of fine-grained cell types using predicted gene expression profiles. To address this, Zhao *et al.* (2024) introduced a one-stage pipeline, to resolve fine-grained cell types from histology images using a graph-transformer framework, while Wu *et al.* (2024) adopted a deep learning method to predict ST-driven cell types and annotate cells solely from image spots. Both methods leveraged histological images to create fine-grained cellular spatial architectures. However, these approaches overlook the molecular information embedded in gene expression data, which is fundamental to defining fine-grained transcriptional cell types. Particularly, they neglect marker genes that are highly expressed in specific cell types and are routinely utilized for cell cluster annotation. These genes play a pivotal role in determining cell identity and function (Ianevski *et al.* 2022). This gap motivates us to explore how to jointly harness paired histology images and key gene expression data during training to provide further hints for the identification of fine-grained cell types in histology.

In this work, we present CUCA, a Cross-modal Unified representation learning framework designed to identify fine-grained Cell types from histology imAges. CUCA is trained on paired morphology-molecule ST data and infers fine-grained transcriptional cell abundances directly from pathology images. A morphology-modal representation module is firstly developed to learn morphological patterns exhibited in spot patch images. Meanwhile, the molecular-modal representation module is introduced to distill key molecular signatures inherent in gene expression data through a gene expression reconstruction task. The cross-modal embedding alignment is designed to harmonize the embedding spaces of the morphological and molecular modalities during training, enhancing interaction between the two and integrating image morphology with molecular gene expression patterns. CUCA was applied to three ST datasets obtained via 10x Genomics Visium technology, comprising: (i) healthy human lung tissue, and (ii) two breast cancer datasets. Experimental results across three datasets highlight that our model improves the capability of predicting cell abundances from histology images and reveals effective cross-modal representations of fine-grained transcriptional cell types.

## 2 Materials and methods

**Figure 1.**
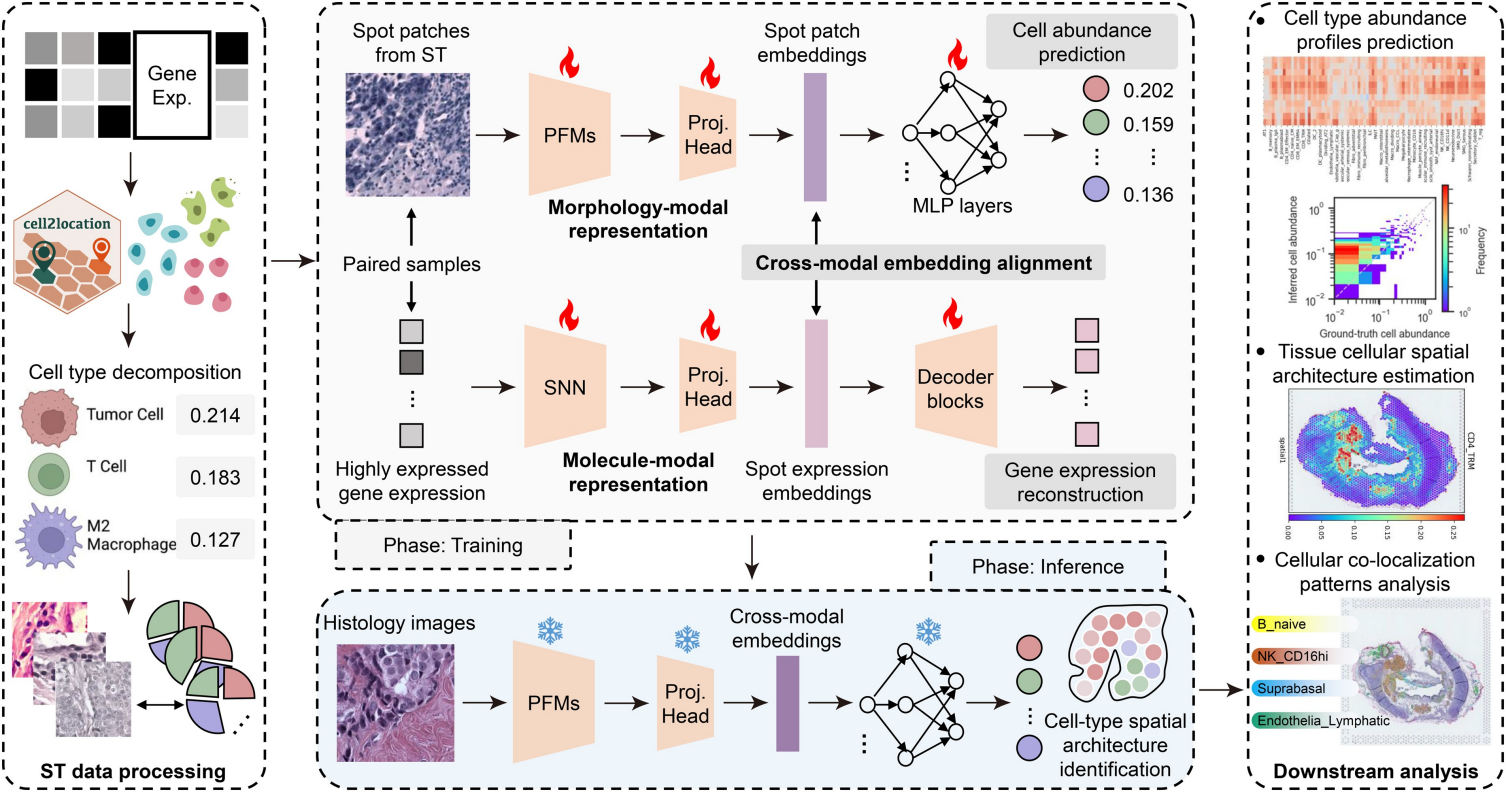
Workflow overview of our framework, CUCA, for cell type identification from histology images via cross-modal unified representation learning. It is composed of (i) ST data processing, (ii) Cross-modal representation learning, and (iii) Downstream analysis.

Figure 1 illustrates an overview of our framework, CUCA. It is composed of (i) ST data processing, which prepares fine-grained cell abundance labels from spot gene expression data, (ii) cross-modal unified representation learning, which jointly integrates paired spot patches with key gene expression data during training, and (iii) downstream analysis, which evaluates the performance of our model across a panel of tasks. We next formulate the problem of the identification of fine-grained cell types and describe our framework in detail.

### 2.1 Problem formulation

We consider a sequencing-based ST dataset $X={\left\{X_{i}\right\}}_{i=1}^{N}$, which comprises *N* samples. Each sample $X_{i}$ contains $n_{i}$ observation spots, represented as ${\left\{x_{j}^{i}\right\}}_{j=1}^{n_{i}}$, where *i* indicates the sample index and *j* indicates the spot index. Each observation spot $x_{j}^{i}$ includes a local histological patch image $p_{j}^{i}\in R^{3\times H\times W}$ and a set of gene expression values $e_{j}^{i}\in R^{1\times d}$, where *H* and *W* indicate the height and width of patch image, and *d* denotes the number of genes. These gene expression values collectively form a spatial gene expression matrix $E_{i}$ for the sample $X_{i}$.

(1) $$E_{i}={\left[e_{0}^{i}{}^{T},e_{1}^{i}{}^{T},\ldots ,e_{n_{i}}^{i}{}^{T}\right]}^{T},\;\;\;E_{i}\in R^{n_{i}\times d}$$

Cell type deconvolution algorithms D(·) commonly decompose fine-grained cell abundances $y_{j}^{i}\in R^{1\times k}$ merely from gene expression data $e_{j}^{i}$ for cell type identification, where *k* is the number of cell types, and it is significantly smaller than the number of genes *d*.

(2) $$Y_{i}\leftarrow D(E_{i}),\;\;\;Y_{i}\in R^{n_{i}\times k},\;k\ll d$$

In the task of identifying fine-grained cell types from histology images, the objective is to learn a function F(·) that predicts cell abundance profiles ${\hat{y}}_{j}^{i}$ from the corresponding spot patch image $p_{j}^{i}$. F(·) is typically parameterized by deep neural networks.

(3) $$\begin{array}{c} {\hat{Y}}_{i}=\left[\begin{array}{c} {\hat{y}}_{0}^{i}\\ {\hat{y}}_{1}^{i}\\ \ldots \\ {\hat{y}}_{n_{i}}^{i} \end{array}\right],\;\;\;{\hat{y}}_{j}^{i}\leftarrow F(p_{j}^{i}),\;\;\;{\hat{y}}_{j}^{i}\in R^{1\times k} \end{array}$$

The key challenge lies in how to jointly harness paired histology patch images $p_{j}^{i}$ and gene expression data $e_{j}^{i}$ to provide rich information and thus achieve precise cell abundance prediction. We here integrate key gene patterns $h_{j}^{i}$ with morphological histology $p_{j}^{i}$ during training:

(4) $${\hat{y}}_{j}^{i}\leftarrow F(p_{j}^{i},h_{j}^{i}),\;\;\;h_{j}^{i}=\psi (e_{j}^{i}),\;\;h_{j}^{i}\subseteq e_{j}^{i}$$

 

(5) $$\psi (\cdot ):\;R^{1\times d}\to R^{1\times r},\;\;\;r\ll d$$

 ψ(·) is capable of discovering key gene molecular information $h_{j}^{i}$, where $h_{j}^{i}$ is the subset of gene expression data $e_{j}^{i}$. *r* represents the number of key genes.

### 2.2 ST data processing

The objective of processing raw ST data is to generate fine-grained cell abundances $y_{j}^{i}\in R^{1\times k}$ using cell type deconvolution methods D(·). To ensure sensitivity, efficiency, and high resolution, we use the publicly available Cell2location method for cell type decomposition and abundance estimation. This approach leverages a reference single-cell transcriptomics atlas alongside ST spot gene expression data. The resulting cell abundance estimates serve as ground truth labels for optimizing our model F(·). Additionally, we retain the top 250 highly expressed genes and extract 224 × 224 pixel patch images from histology WSIs at 20× magnification, with the images centered on the corresponding ST spots. This process produces a comprehensive dataset comprising highly expressed gene expression data $h_{j}^{i}$, patch images $p_{j}^{i}$, and cell abundance labels $y_{j}^{i}$.

(6) $$X_{i}\leftarrow {\left\{{\tilde{x}}_{j}^{i}\right\}}_{j=1}^{n_{i}}=\{(h_{0}^{i},p_{0}^{i},y_{0}^{i}),\ldots ,(h_{n_{i}}^{i},p_{n_{i}}^{i},y_{n_{i}}^{i})\}$$

### 2.3 Cross-modal unified representation learning

Cross-modal unified representation learning includes three key components. Morphology-modal representation derived from spot patch images, Molecule-modal representation based on highly expressed gene expression data, and Cross-modal embedding alignment for integration of morphological histology with molecular gene patterns. It is noteworthy that this component is exclusively applied during model training.

#### 2.3.1 Morphology-modal representation

The morphology-modal representation branch, denoted as $R_{\mathrm{morph}}(\cdot )$, is designed to extract morphological features directly from ST patch images. Recent advancements in pathology foundation models (PFMs), pretrained on large-scale datasets of histopathological whole-slide images (WSIs), have significantly advanced the field of computational pathology (Xu *et al.* 2024, Zimmermann *et al.* 2024). We thus explore PFMs as the backbone, T(·), and fine-tune them to capture diverse cellular and tissue patterns using ST patch image data. Specifically, the spot images $p_{j}^{i}$, with sizes of 224 × 224 pixels, serve as input for this branch. They undergo representation learning to extract relevant morphological features using fine-tuned PFMs $\tilde{T}(\cdot )$. The PFMs are efficiently fine-tuned using the Low-Rank Adaptation of Large Language Model (LoRA) method (Hu *et al.* 2022). The extracted features are then processed through a projection head P(·) for nonlinear transformation, producing morphology-modal embeddings $g_{j}^{i}$ with dimensionality $d_{a}$.

(7) $$g_{j}^{i}=R_{\mathrm{morph}}(p_{j}^{i})=P(\tilde{T}(p_{j}^{i})),\;\;\;g_{j}^{i}\in R^{1\times d_{a}}$$

These embeddings capture representations of histology images and hold promise for integration with molecular data.

#### 2.3.2 Molecule-modal representation

The molecule-modal representation branch, denoted as $R_{\mathrm{molec}}(\cdot )$, is introduced to learn molecular patterns from ST gene expression data corresponding to the spot patch images. To enhance the expressiveness of molecular feature representations, we use a Self-Normalizing Network (SNN) (Chen *et al.* 2021) for molecular representation learning. The highly expressed gene data $h_{j}^{i}$ is processed through an optimized SNN, denoted as $\tilde{S}(\cdot )$, followed by a projection head H(·) that maps the features into embeddings for the molecule modality. These embeddings are referred to as spot expression embeddings $s_{j}^{i}$, with a dimensionality of $d_{a}$.

(8) $$s_{j}^{i}=R_{\mathrm{molec}}(h_{j}^{i})=H(\tilde{S}(h_{j}^{i})),\;\;\;s_{j}^{i}\in R^{1\times d_{a}}$$

#### 2.3.3 Cross-modal embedding alignment

Using the morphology-modal patch embeddings $g_{j}^{i}$ and molecule-modal expression embeddings $s_{j}^{i}$, we develop the cross-modal embedding alignment module C(·) to integrate gene expression patterns with histology features. We design a cross-modal consistency alignment loss $L_{C}(\cdot )$ to align the embedding spaces of both modalities, which unifies the underlying signatures of morphological histology and molecular expression. Formally, for a batch of *B* paired morphology-modal patch embeddings and molecule-modal expression embeddings ${\left\{(g_{m},s_{m})\right\}}_{m=1}^{B}$ from the sample ${\left\{(p_{m},h_{m},y_{m})\right\}}_{m=1}^{B}$, the objective is defined as:

(9) $$L_{C}(\cdot )=\frac{1}{\sqrt{B}}{\left(\sum_{m=1}^{B}{\left\|g_{m}-s_{m}\right\|}_{2}\right)}^{\frac{1}{2}}$$

Here, $||\cdot |{|}_{2}$ denotes the L2 norm. This function aims to minimize the absolute distance between paired morphology-modal and molecule-modal embeddings derived from the same sample.

To establish an end-to-end training framework for cross-modal embedding alignment and cell type prediction, we utilize cell abundance labels $y_{m}$ as supervised signals to learn cellular patterns from histology images $p_{m}$. Specifically, the aligned embeddings ${\tilde{g}}_{m}$ from morphology modality are fed into a multilayer-perceptron (MLP) block A(·) to predict cell abundance profiles. This process linearly transforms the aligned spot patch embeddings ${\tilde{g}}_{m}$ into predicted abundance values ${\hat{y}}_{m}$ for cell types. The loss function $L_{A}(\cdot )$ is defined as:

(10) $$\begin{array}{c} {\hat{y}}_{m}=A({\tilde{g}}_{m}),\;\;\;{\hat{y}}_{m}\in R^{1\times k}\\ L_{A}(\cdot )=\frac{1}{\sqrt{B}}{\left(\sum_{m=1}^{B}{\left\|y_{m}-{\hat{y}}_{m}\right\|}_{2}\right)}^{\frac{1}{2}} \end{array}$$

Given that the cell abundance labels $y_{m}$ are continuous, we use a regression-based strategy and apply the Root Mean Squared Error (RMSE) loss as the objective function to optimize the model for predicting cell abundance profiles.

Furthermore, we introduce a gene expression reconstruction task to enhance the model’s ability to distill molecular information inherent in gene expression data for embeddings $s_{m}$. The aligned spot expression embeddings ${\tilde{s}}_{m}$ from the molecule modality are fed into the decoder block G(·) to reconstruct gene expression patterns, leveraging the learned cross-modal molecular representations ${\tilde{s}}_{m}$.

(11) $$\begin{array}{c} {\hat{h}}_{m}=G({\tilde{s}}_{m}),\;\;\;{\hat{h}}_{m}\in R^{1\times r}\\ L_{G}(\cdot )=\frac{1}{\sqrt{B}}{\left(\sum_{m=1}^{B}{\left\|h_{m}-{\hat{h}}_{m}\right\|}_{2}\right)}^{\frac{1}{2}} \end{array}$$

where ${\hat{h}}_{m}$ indicates the reconstructed gene expression values, and $L_{G}(\cdot )$ denotes the gene expression reconstruction loss function. This approach captures molecular information to enrich histology representation, facilitating the identification of fine-grained cell types from histology images.

### 2.4 Overall loss optimization

The overall loss objective L(·) is formulated as the weighted sum of three loss objective terms.

(12) $$\begin{array}{c} L(\cdot )=\lambda_{C}\cdot L_{C}(\cdot )+\lambda_{A}\cdot L_{A}(\cdot )+\lambda_{G}\cdot L_{G}(\cdot )\\ \left\{{\tilde{\theta }}_{\mathrm{morph}},{\tilde{\theta }}_{\mathrm{molec}},{\tilde{\theta }}_{A},{\tilde{\theta }}_{G}\}\leftarrow \arg\min_{\left\{\theta_{\mathrm{morph}},\theta_{\mathrm{molec}},\theta_{A},\theta_{G}\right\}}L(\cdot \right) \end{array}$$

where $\lambda_{C}$, $\lambda_{A}$, and $\lambda_{G}$ denote the balanced weights for the cross-modal consistency alignment loss $L_{C}(\cdot )$, cell abundance prediction loss $L_{A}(\cdot )$, and gene expression reconstruction loss $L_{G}(\cdot )$, respectively. $\theta_{\mathrm{morph}}$, $\theta_{\mathrm{molec}}$, $\theta_{A}$, and $\theta_{G}$ are the parameters in $R_{\mathrm{morph}}(\cdot )$, $R_{\mathrm{molec}}(\cdot )$, A(·), and G(·). The term argmin(·) aims to find the optimized parameters $\tilde{\theta }$ that minimize the overall loss function L(·). It is worth noting that an L2 regularization term is added to the objective L(·) through the optimizer.

### 2.5 Inference

During inference, the histology image is processed through the fine-tuned PFMs encoder and projection head to extract cross-modal embeddings, which capture morphological patterns enhanced by molecular gene expression information. The resulting embeddings are then transformed into predicted fine-grained cell abundances using a MLP regression head. The predicted cell abundances, along with the corresponding spot coordinates, are used to create the cellular spatial architecture and analyze co-localization patterns among cell types.

## 3 Experiments and results

### 3.1 Data description

The **humanlung** dataset (Madissoon *et al.* 2023) comprises 20 770 Visium spots from healthy human lung tissue, with matched H&E-stained WSIs and ST data. The her2st (Andersson *et al.* 2021) and stnet (He *et al.* 2020) datasets include 13 620 and 30 655 Visium spots, respectively, from HER2-positive breast cancer and general breast cancer tissues, each paired with H&E and gene expression data. Cell2location (v0.1) was applied to estimate fine-grained cell type abundances for all datasets, with reference information and data details provided in the Supplementary Notes S1.

### 3.2 Experimental designs and comparative methods

Implementation details, data splits, and evaluation criteria can be found in the Supplementary Notes S2 and S3. Our model was compared against state-of-the-art methods, including Hist2Cell (Zhao *et al.* 2024), ST-Net (He *et al.* 2020), THItoGene (Jia *et al.* 2023), and HisToGene (Pang *et al.* 2021). Brief introductions to these methods are provided in Supplementary Notes S4. Additionally, the model that utilized PFMs combined with the linear probing strategy, denoted as Linearprobing, was implemented for comparison.

### 3.3 CUCA enhances cell abundance prediction capability

**Figure 2.**
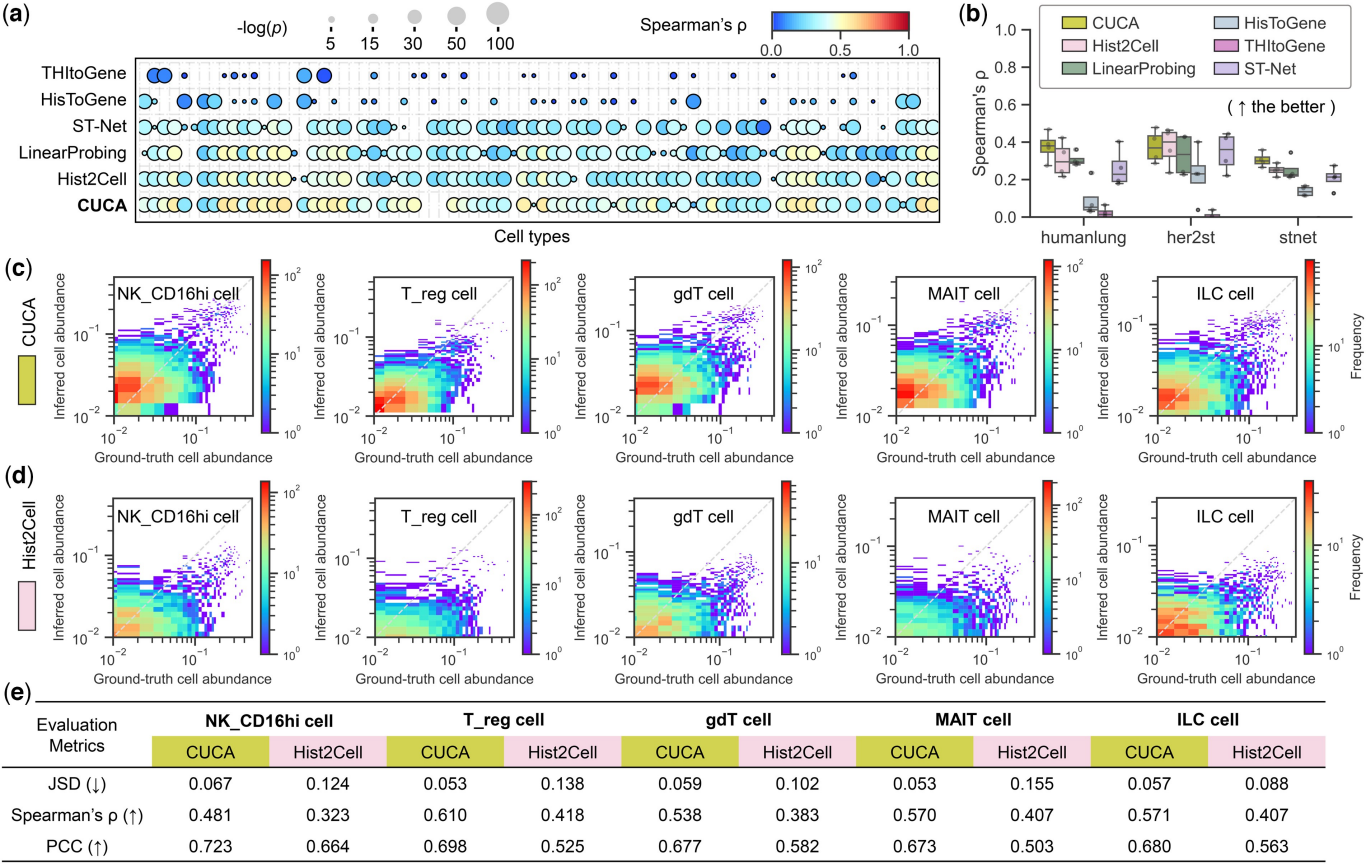
Performance evaluation of fine-grained cell abundance prediction across methods. (a) Dot plot comparing 80 cell types in the humanlung dataset, with color indicating Spearman’s ρ and size representing the negative logarithm of the *P*-value (two-sided *t*-test approximation). (b) Split-level averaged Spearman’s ρ performance on the humanlung, her2st, and stnet datasets. (c and d) Log-scale 2D histograms of predicted versus ground truth abundances for key cell types in (c) CUCA and (d) Hist2Cell, with the first diagonal (*y* = *x*) reference line and color-coded 2D histogram counts. (e) Quantitative evaluation results for each cell type in (c): JSD (Jensen–Shannon Divergence), Spearman’s ρ, and PCC (Pearson correlation coefficient).

To evaluate the effectiveness of our model, we conducted a systematic comparison of CUCA’s performance against state-of-the-art methods. The results demonstrate that CUCA outperformed existing approaches in predicting fine-grained cell abundance profiles from histology images, as evidenced by higher Spearman’s rank (Spearman’s ρ) correlation coefficients and Jensen–Shannon Divergence (JSD) values (Fig. 2a and b, Supplementary Figs S3 and S4, and Supplementary Table S1–S3). The predicted cell abundances exhibited positive correlations with the ground truth labels estimated by Cell2location, with statistically significant positive Spearman’s ρ correlations observed for 69 of 80 cell types in the humanlung dataset (Fig. 2a), 38 of 39 cell types in the her2st dataset, and all 39 cell types in the stnet dataset (Supplementary Fig. S3a and b). CUCA showed notable improvements of 22.5% and 21.9% in Spearman’s ρ values across the humanlung and stnet datasets, compared to the second-best method (Fig. 2b and Supplementary Table S2). CUCA also exhibited a stronger correlation with ground truth labels compared to other methods, as measured by the JSD metric. It achieved an average JSD value of 0.166 across all 80 fine-grained cell types in the humanlung dataset and presented 0.121 and 0.103 across 39 cell types in the her2st and stnet datasets, respectively (Supplementary Fig. S4 and Supplementary Table S1). Considering variability across tissue slices, we evaluated our model on individual slides. We can observe that CUCA outperformed these comparative methods in terms of a series of evaluation metrics, including the JSD, Spearman’s ρ, and Pearson correlation coefficient (PCC), demonstrating the superior performance in prediction consistency among samples. (Supplementary Figs S5 and S6 and Supplementary Table S4–S6). These results indicate that CUCA is capable of predicting fine-grained cell abundances from histology images, highlighting the advantage of the proposed cross-modal learning framework and the molecule-morphology embedding alignment paradigm.

In particular, several cell types demonstrated predictable performance (Fig. 2c–e and Supplementary Figs S7 and S8). We highlighted Natural killer CD16 high expressed (NK_CD16hi) cells, Regulatory T (T_reg) cells, Gamma-delta T (gdT) cells, Mucosal-associated invariant T (MAIT) cells, and Innate lymphoid cells (ILC) from the humanlung dataset. The cell abundances inferred by CUCA exhibited positive correlations with the ground truth labels (Fig. 2c), with Spearman’s ρ exceeding 0.45 for these cell types (Fig. 2e), outperforming the second-best method, hist2cell (Fig. 2d). These predictable cell types play crucial roles in the tumor system. For instance, NK_CD16hi cells are essential for tumor immune surveillance and immunotherapy, inhibiting the growth and spread of tumor cells through its antibody-dependent cellular cytotoxicity and direct killing effect (Page *et al.* 2024). Similarly, MAIT cells are significant in tumor immunity, with tumor-infiltrating MAIT cells potentially serving as positive prognostic factors (Godfrey *et al.* 2019). These findings underscore CUCA’s ability to integrate key gene expression patterns with histology embeddings, enabling improved prediction of fine-grained cell types that are crucial for understanding tumor biology and advancing cancer research.

### 3.4 CUCA resolves cellular spatial architectures

**Figure 3.**
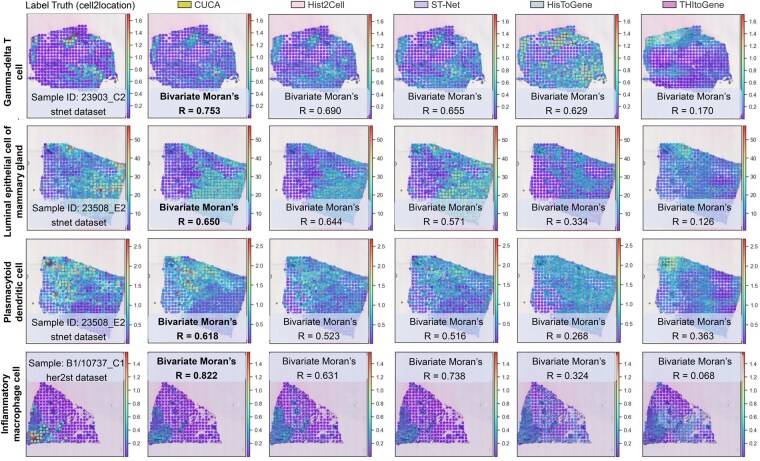
Spatial distribution of predicted cell abundances for key fine-grained cell types, with color scales aligned to that of label truth for each cell type. Bivariate Moran’s *R* index, measuring spatial correlation, is displayed for each plot. Color bar represents cell abundance levels.

We examined the spatial distribution maps of fine-grained cell types using spot spatial coordinates and the cell abundances predicted by CUCA and comparative methods. We assessed the spatial correlation between Cell2location-estimated cell abundances and model predictions using bivariate Moran’s R index. CUCA showed superior consistency with the true cell type spatial distributions estimated by Cell2location, capturing cellular spatial architectures better than other methods, as evidenced by bivariate Moran’s R evaluation (Fig. 3 and Supplementary Figs S9–S11). In breast cancer samples, CUCA resolved key cellular spatial architectures, achieving bivariate Moran’s R values exceeding 0.6 for several cell types, including gdT cells, luminal epithelial cells of the mammary gland, class-switched memory B cells, regulatory T cells, and plasmacytoid dendritic cells (Fig. 3). It showed the ability to capture both local patterns, such as cell density, and global characteristics, including cell localization trends. For the humanlung dataset, CUCA captured the spatial distributions of CD8 positive tissue-resident effector memory T (CD8_TRM_EM) cells, NK_CD16hi cells, myoepithelial cells, CD8_TRM cells, and ciliated cells, even in locally aggregated and sparse tissue regions (Supplementary Fig. S9). In contrast, the spatial architectures of CD8_TRM_EM, myoepithelial, and NK_CD16hi cells predicted by Hist2Cell appeared ambiguous, making it challenging to discern architecture patterns of specific cell types. The observations indicate that our method is capable of resolving fine-grained cellular spatial architectures from histology images, offering potential insights into tissue organization and cell arrangement.

### 3.5 CUCA elucidates cell-type co-localization patterns

**Figure 4.**
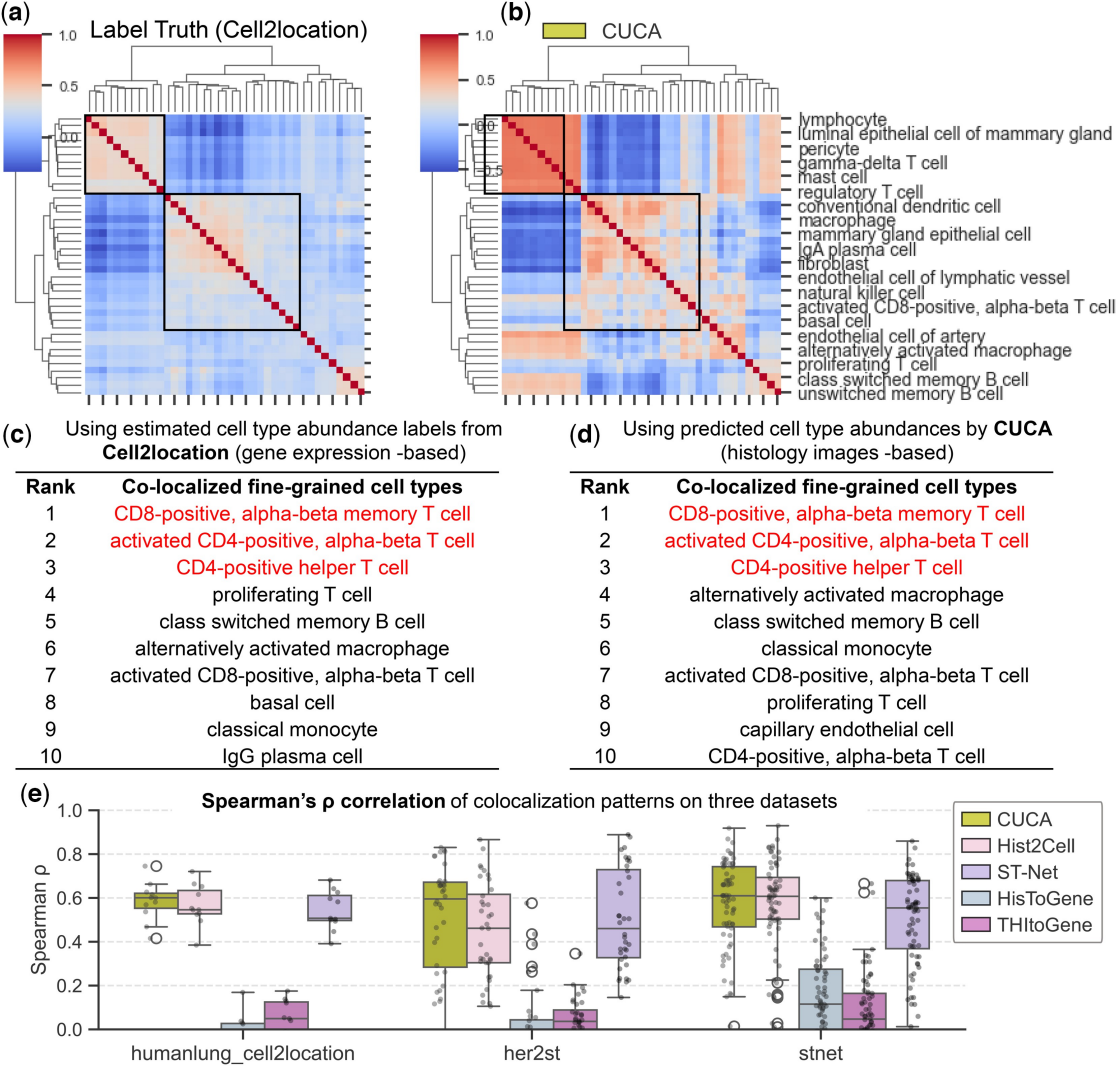
Analysis of fine-grained cell type co-localization patterns. (a and b) Hierarchically clustered heatmaps of spatial correlations (bivariate Moran’s *R*) between cell type pairs in stnet dataset: (a) ground truth and (b) CUCA (key patterns boxed). Color bar represents bivariate Moran’s R levels between cell type pairs. (c and d) Key co-localized cell types of CD4${}^{+}$_EM_T cells in breast cancer identified by (c) Cell2location and (d) CUCA. (e) Spearman’s ρ performance comparing predicted versus ground truth global co-localization patterns across three datasets.

We investigated the co-localization patterns among fine-grained cell types in human lung and breast tissues. We evaluated the correlation between the abundances of paired cell types while accounting for spatial auto-correlation. Hierarchically clustered heatmaps illustrating the co-localization patterns for ground truth labels, CUCA predictions, and comparative methods across the three datasets are shown in Fig. 4a and b and Supplementary Figs S12–S14. Our method demonstrated the ability to capture the spatial co-localization interaction patterns among 39 fine-grained cell types in breast cancer samples. The observed co-localization patterns from CUCA suggested a trend of consistency with the patterns derived from Cell2location (Fig. 4a and b). In contrast, HisToGene and THItoGene displayed discrepancies compared to the ground truth patterns (Supplementary Fig. S12d and e). This presents the limitations of existing methods in discovering the spatial co-localization patterns among fine-grained cell types. Notably, CUCA recognized key co-localizations between CD4${}^{+}$_EM_T cells and various T cell subtypes in breast cancer samples from the stnet dataset (Fig. 4c and d). The top three co-localized cell types with CD4${}^{+}$_EM_T cells, including CD8-positive alpha-beta memory T cells, activated CD4-positive alpha-beta T cells, and CD4-positive helper T cells, were consistent between Cell2location ground truth labels and CUCA predictions. These T cell co-localization patterns contribute to the potential responses to cancer immunotherapy in breast cancer (Schenkel and Pauken 2023), highlighting the importance of studying cell type interactions within the TME (Ruan *et al.* 2024b).

We further quantified the Spearman’s ρ correlation and cosine similarity between the global co-localization patterns derived from ground truth labels and those obtained from each method. CUCA showed higher Spearman’s ρ performance across three datasets, compared to other methods (Fig. 4e). It also indicated improvements in measuring the cosine similarity of co-localization patterns (Supplementary Fig. S15). These analyses underscore the effectiveness of CUCA in elucidating key interactions and global co-localization patterns among fine-grained cell type pairs.

### 3.6 CUCA ablation experiments

To explore the components of our proposed cross-modal unified representation learning framework, we conducted ablation experiments focusing on four key aspects. (i) Pathology foundation models, (ii) cross-modal embedding alignment, (iii) sensitivity analysis of weights on modalities, (iv) impact of loss functions, and (v) impact of latent embedding dimensions. Details are provided in Supplementary Notes S5 and S6.

####  3.6.1 Pathology foundation models

**Figure 5.**
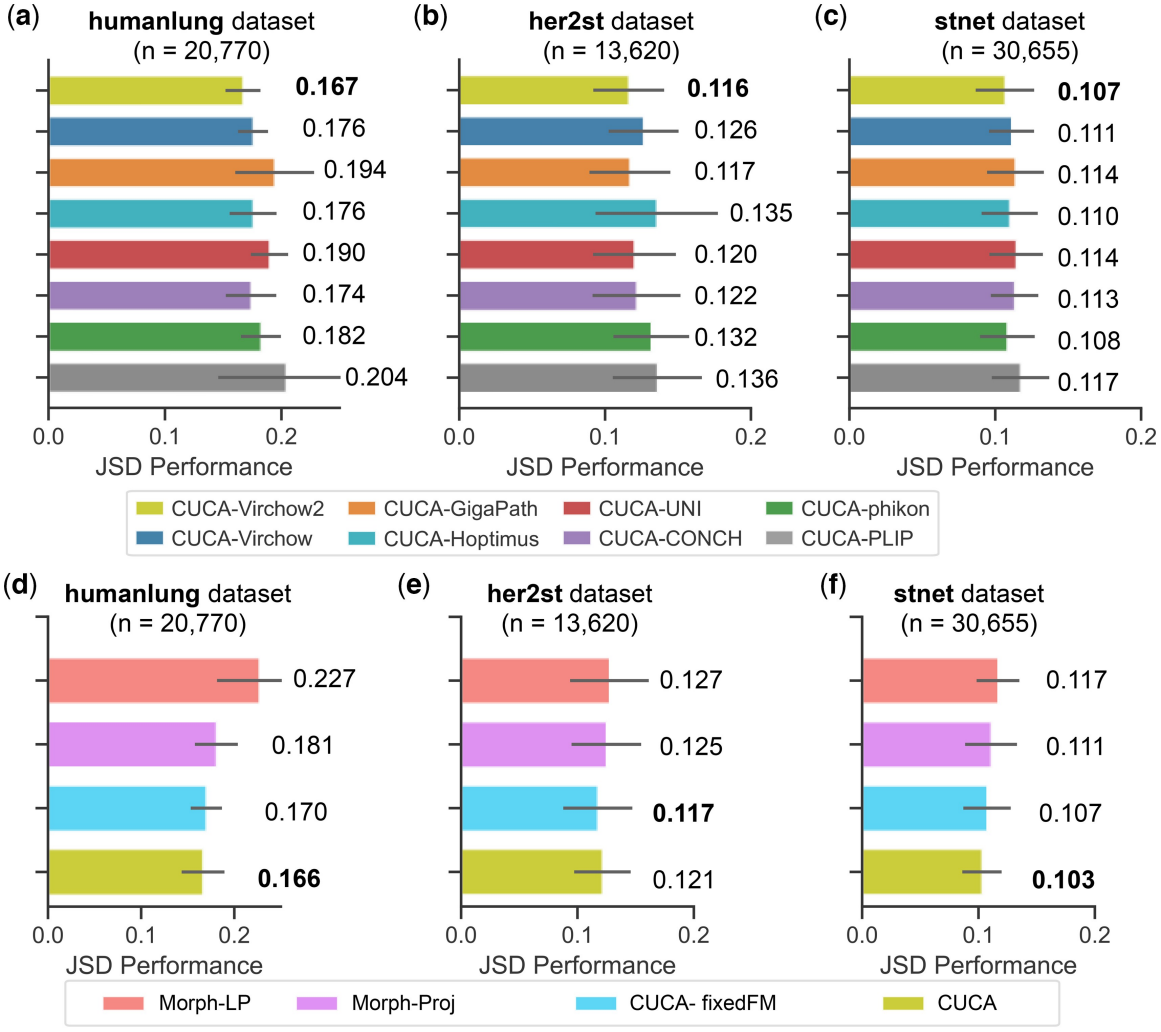
Ablation study of pathology foundation models (a and c) and cross-modal embedding alignment (d and f) across three datasets: (a, d) humanlung, (b, e) her2st, and (c, f) stnet. Mean JSD values are shown on the plot with standard deviation indicated by error lines. Best performance is highlighted in *bold*.

We evaluated the performance of CUCA using different patch-level PFMs. The Virchow2 model (Zimmermann *et al.* 2024) achieved superior performance across the humanlung, her2st, and stnet datasets (Fig. 5a–c and Supplementary Fig. S16). Notably, all self-supervised PFMs consistently outperformed comparative methods on the humanlung dataset, achieving JSD performance ranging from 0.204 to 0.167 (Fig. 5a). These results indicate the significance of leveraging robust PFMs to yield morphological representations of histological tissues. Consequently, we used Virchow2 for all subsequent analyses.

#### 3.6.2 Cross-modal embedding alignment

We developed ablated models that utilized PFMs on morphological images (details provided in Supplementary Notes S5). We can observe that Morph-Proj outperformed Morph-LP across three datasets (Fig. 5d–f and Supplementary Fig. S17) and CUCA demonstrated better performance compared to CUCA-fixedFM model in the humanlung and stnet datasets. This implies that fine-tuning PFMs using ST data enhances the transformation of spot patch images into an informative feature space. Furthermore, cross-modal CUCA outperformed the morphology-only Morph-Proj model, improving Spearman’s ρ by 10.6% and 7.4% across both breast cancer datasets, respectively (Supplementary Fig. S17). These results reinforce that incorporating molecular expression data enhances the identification of fine-grained cell types from histology images, highlighting the effectiveness of our cross-modal embedding alignment.

#### 3.6.3 Sensitivity analysis of weights on modalities

We conducted experiments by adjusting balanced weights of loss functions for morphology-modal and molecule-modal representations. From our observations, JSD performance remained relatively stable as the weight ratio of the morphology to molecule modalities increased (Supplementary Fig. S18a). Reducing the weight on the molecule modality led to a slight decline in correlation performance on the humanlung and stnet datasets, while the her2st dataset showed improved performance under these conditions (Supplementary Fig. S18b–c). We attribute this observation to dataset size, as larger datasets are more likely to benefit from the inclusion of the molecular information. These results reveal that the molecular expression reconstruction in our design enhances the model’s ability to predict cell abundances from histology images. Balancing the contributions of morphology and molecule modalities is also crucial for optimizing cross-modal models.

#### 3.6.4 Impact of loss functions

We implemented RMSE and MSE as target loss functions for global optimization. We also compared Kullback–Leibler divergence (KLD) and contrastive information noise contrast estimation (infoNCE) (Stärk *et al.* 2022, Lu *et al.* 2024) loss functions for cross-modal embedding alignment. The model optimized with L2 distance outperformed the one using mutual information-based infoNCE (Supplementary Fig. S19). The RMSE-based model also excelled in aligning histological and molecular embeddings compared to the models using KLD and MSE loss functions. These findings indicate that RMSE is effective in reflecting the magnitude of prediction errors and minimizing the absolute distance between paired multi-modal embeddings.

### 3.7 CUCA manifests cross-modal patterns of cell types

**Figure 6.**
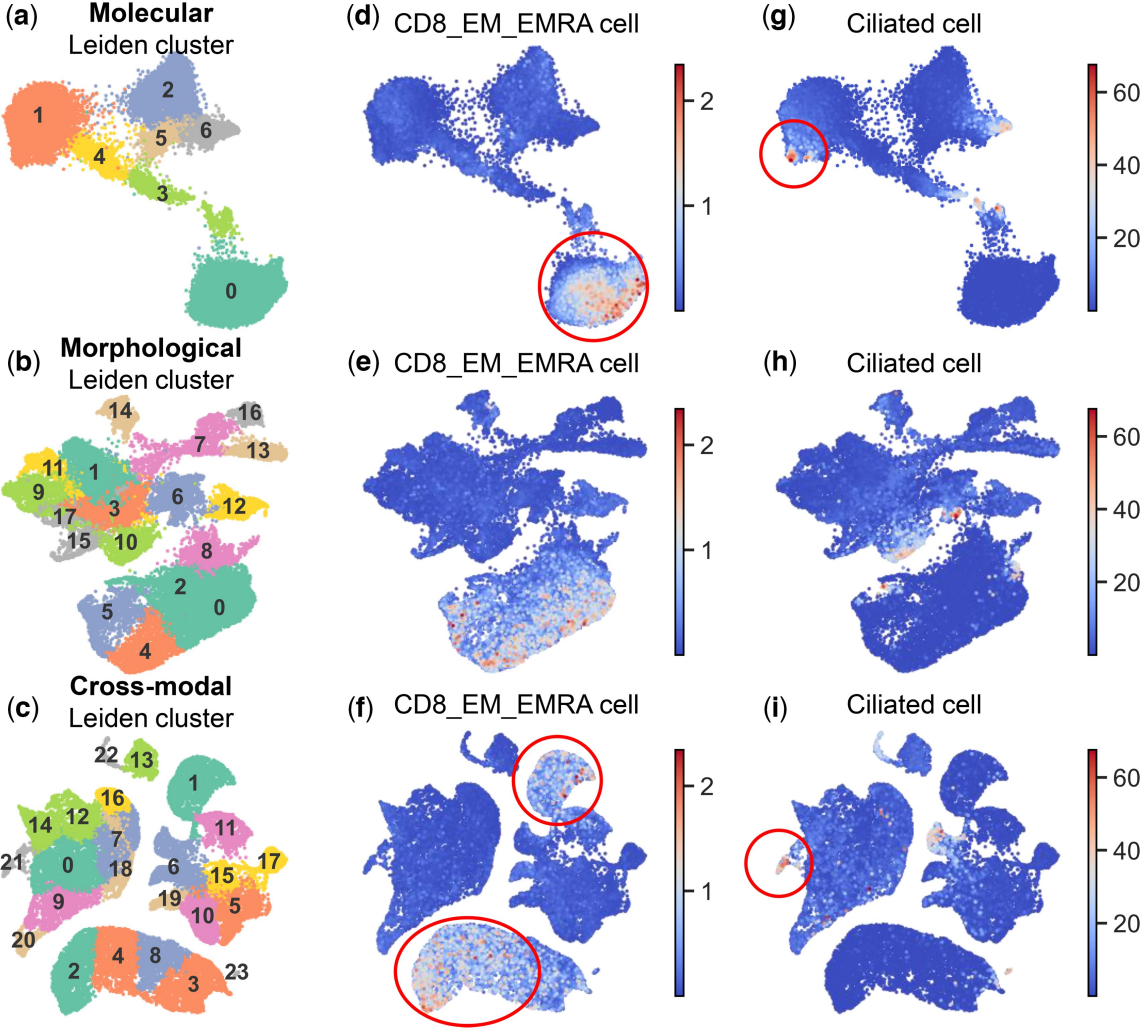
UMAP visualizations of humanlung ST sample embeddings: (a and c) Cluster distributions for (a) molecular, (b) morphological, and (c) cross-modal embeddings (colors=cluster IDs). (d–i) Cell-type-specific patterns for (d–f) CD8_EM_EMRA and (g–i) ciliated cells, with color intensity reflecting cell abundance.

We visualized the low-dimensional embeddings of molecule-only, morphology-only, and morphology-molecule cross-modal data across spots in humanlung ST samples using Uniform Manifold Approximation and Projection (UMAP). Leiden clustering was performed to characterize the diversity of local spots. We identified 7 subclusters from the molecular gene expression data and 18 subclusters from the paired histology images. In contrast, the cross-modal embeddings yielded 24 subclusters (Fig. 6a–c), revealing greater diversity compared to the morphology-only and molecule-only embeddings. Notably, the 24 cross-modal subclusters formed well-separated subgroups, aligning with the expression levels of key genes (Supplementary Fig. S20). This implies that the cross-modal embeddings may integrate morphological patterns with key molecular expression, potentially uncovering distinct phenotypic subtypes.

We further analyzed the distribution of fine-grained cell types within the embeddings. Key cell types, including CD8_EM_EMRA, Ciliated, Muscle_airway, and SMG_Mucous cells, were visualized (Fig. 6d–i). CD8_EM_EMRA cell type primarily aligned with subcluster 0 in the molecule-only embeddings but was distributed across subcluster 1, 2, 4, and 8 in the cross-modal embeddings, demonstrating the benefits of integrating morphological patterns. Additionally, a subset of spots in the molecule-modal subcluster 1 exhibited high abundances of Ciliated cells, which was marked as an individual subcluster 21 in the cross-modal UMAP. Comparable patterns were observed for Muscle_airway and SMG_Mucous cell types. These results highlight how cross-modal alignment between morphology and molecule data can facilitate the discovery of phenotypes associated with fine-grained cell types. More results can be found in Supplementary Figs S21–S23.

## 4 Conclusion

In this work, we present CUCA, a cross-modal unified representation learning framework designed to predict fine-grained cell types from histology images. CUCA leverages pathology foundation models to enhance morphological representations of histological patches in ST data and incorporates a gene expression reconstruction task to distill key molecular signatures from gene expression data during training. The proposed cross-modal embedding alignment integrates image morphology with molecular gene expression patterns, enabling improved prediction of fine-grained cell abundance profiles compared to existing methods. Experimental results demonstrate that CUCA achieves remarkable performance improvements over existing methods across three ST datasets. Moreover, CUCA shows the ability to resolve fine-grained cellular spatial architectures and uncover co-localization interaction patterns among cell types, providing potential insights into intercellular spatial characterization within tumor ecosystems. Future work will focus on improving the method’s robustness to more datasets and extending its application to additional tissue types and external clinical pathology cohorts.

## Supplementary Material

btaf201_Supplementary_Data

## Data Availability

All the data used in this manuscript are publicly available. The healthy lung dataset derived from the integrated Human Lung Cell Atlas can be obtained from the public domain: https://5locationslung.cellgeni.sanger.ac.uk/. The her2st data is available in Zenodo, at https://doi.org/10.5281/zenodo.4751624. The STNet data is available in Mendeley Data at https://data. mendeley.com/datasets/29ntw7sh4r/5. The source code of CUCA is available in GitHub, at github.com/lyotvincent/CUCA.
