# Supplementary material for: Predicting fine-grained cell types from histology images through cross-modal learning in spatial transcriptomics
Source: Bioinformatics. 2025 Jul 15;41(Suppl 1):i115–24. doi: 10.1093/bioinformatics/btaf201 (PMC12261428; doi:10.1093/bioinformatics/btaf201)
Supplement: btaf201_Supplementary_Data [file btaf201_supplementary_data.zip › btaf201_Supplementary_Data/Liu.78.ALT TEXT File.pdf]

the alt-text for each figure in our paper *Predicting fine-grained cell types from histology images through cross-modal learning in spatial transcriptomics*

Figure 1

Diagram of CUCA framework: ST data processing, cross-modal learning, and downstream analysis for cell type identification from histology images.

Figure 2

Graphs comparing cell abundance prediction performance across methods using dot plots, 2D histograms, and quantitative evaluation metrics.

Figure 3

Images showing spatial distribution of predicted cell abundances with color-coded scales and Bivariate Moran's R for spatial correlation.

Figure 4

Graphs and data showing cell type co-localization patterns and spatial correlation comparisons across methods and datasets.

Figure 5

Plots showing ablation study results on foundation models and embedding alignment with mean JSD and error bars across datasets.

Figure 6

UMAP plots showing clustering and cell-type-specific abundance patterns from molecular, morphological, and crossmodal embeddings in humanlung data.
