# Supplementary material for: Predicting fine-grained cell types from histology images through cross-modal learning in spatial transcriptomics
Source: Bioinformatics. 2025 Jul 15;41(Suppl 1):i115–24. doi: 10.1093/bioinformatics/btaf201 (PMC12261428; doi:10.1093/bioinformatics/btaf201)
Supplement: btaf201_Supplementary_Data [file btaf201_supplementary_data.zip › btaf201_Supplementary_Data/Liu.78.sup.1.pdf]

Supplementary Materials

Predicting fine-grained cell types from histology images through cross-modal learning in spatial transcriptomics

Chaoyang Yan[1,2], Zhihan Ruan[1,2], Songkang Chen[1,2], Yichen Pan[1,2], Xue Han[1,2], Yuanyu Li[1,2], and Jian Liu[3,*]

Affiliations

1. College of Computer Science, Nankai University, Tianjin, China
2. Centre for Bioinformatics and Intelligent Medicine, Nankai University, Tianjin, China.
3. State Key Laboratory of Medicinal Chemical Biology, College of Computer Science, Nankai University, Tianjin, China

[*] **Corresponding author.** Jian Liu (jianliu@nankai.edu.cn)

This file includes:

Supplementary Notes S1 to S6, Supplementary Tables S1 to S6, Supplementary Figures S1 to S24.

Supplementary Notes

S1. Data description and Cell2location reference signatures

The **humanlung** dataset (Madissoon et al. 2023) was derived from a spatially resolved atlas of the healthy human lung, consisting of 20,770 Visium spots across 11 tissue slices from 4 donors. The dataset includes matched H&E-stained whole-slide images (WSIs) captured at 20× magnification alongside Visium spatial transcriptomics (ST) data collected at 10× resolution. Cell type proportions were estimated using Cell2location (v0.1), leveraging the scRNA-seq reference signature to generate 80 fine-grained transcriptional cell abundance profiles per spot. The reference for deconvolution was constructed from a comprehensive single-cell atlas of the human lung (193,108 cells) (Madissoon et al. 2023), encompassing diverse cell states such as alveolar epithelial subtypes, major immune populations, and stromal components.

The **her2st** dataset (Andersson et al. 2021) originates from a study profiling HER2-positive breast cancer, comprising 13,620 ST spots across 36 tissue sections from 8 patients. The dataset integrates H&E-stained histology slides imaged at 20× magnification (performed using the Metafer VSlide system) with corresponding ST gene expression data. For cell type deconvolution, Cell2location was applied using the Human Breast Cell Atlas (HBCA) (Kumar et al. 2023) as the reference (714, 331 cells), which provides a detailed map of breast tissue heterogeneity, including tumor, immune, and stromal compartments. This process yielded 39 distinct cell type abundance profiles per spot, serving as ground truth labels for model training and evaluation.

Similarly, the **stnet** dataset (He et al. 2020) includes 30,655 spots from 68 breast tissue sections across 23 breast cancer patients, featuring paired H&E slides and ST data. The dataset was processed following established spatial transcriptomics workflows (Ståhl et al. 2016), with Cell2location employed to infer cell type compositions. The HBCA reference was utilized, enabling the resolution of 39 cell states per spot.

In summary, the **humanlung** dataset relies on a lung-specific scRNA-seq reference for deconvolution, while the **her2st** and **stnet** datasets employ the HBCA to resolve breast tissue heterogeneity. All references were selected to maximize biological relevance and granularity in cell type quantification.

S2. Implementation details

Our CUCA model was implemented using Python 3.9.20 and the PyTorch 2.4.1 deep learning framework. The environments for experiments conducted were configured into a docker image (DockerHub: charing/cuca_env), the Dockerfile is provided in our repository (https://github.com/lyotvincent/CUCA). All models, including comparative methods, were conducted on one workstation with two NVIDIA RTX4090 GPUs (24GB×2), the CUDA version is 12.1 and the GPU driver version is 535.171.

The model was trained for 100 epochs with the minibatch size of 128. Adam optimizer was employed for weight parameters optimization and the weight decay was 0.0001. L2 regularization was implemented through the weight_decay in the Adam optimizer. This is equivalent to adding an L2 penalty term to the objective function for all trainable parameters (L2 Weight Decay). This L2 regularization was adopted to mitigate overfitting risks. The learning rate was initially set to 0.0002 and OneCycleLR learning rate policy (Smith and Topin 2019) was applied, which anneals the learning rate from an initial learning rate 0.0002 to maximum

learning rate 0.002 and then from that maximum learning rate 0.002 to minimum learning rate 0.00002. OneCycleLR learning rate policy changes the learning rate after every epoch based on the cosine annealing strategy. The percentage of the cycle (in number of epochs) spent increasing the learning rate was 0.2, which means that the learning rate increases in the first 20 epochs, and the learning rate decreases in the remaining 80 epochs.

The weights of all layers in our model were initialized with the Kaiming uniform strategy. During the training phase, the model with the best Pearson correlation coefficient (PCC) value on the validation set was recorded and utilized for the following evaluation on the test set.

For the three datasets, 224×224 image patches centered on spots in spatial transcriptomics (ST) data was cropped from the whole-slide histology images. The preprocessing of these image patches and corresponding gene expression matrix data was conducted following the strategy used in (Zhao et al. 2024).

Specifically, for the her2st and stnet datasets, we employed a 4-fold cross validation scheme. Each ST dataset was split into four folds, with samples from the same patient in the same fold. In each experiment, one fold was used as the test set and the remaining three folds were mixed. We randomly divided one-third of the patients from this mixed set for validation while the remaining patients were used for model training. The best model performed best in the validation set was chosen to evaluate patients in this test set (fold). This process was repeated for four folds, ensuring that each fold served as the test set exactly once. The average results over the four-fold experiments were reported. The same 4-fold splits were used for each comparative method to ensure fair comparison. For the humanlung dataset, which includes 4 cross-validation splits with training and validation set in the repository of Hist2Cell (Zhao et al. 2024), we employed the leave-one-out cross-validation, where we iteratively trained our model on three of the donor samples and made predictions on the remaining held-out donor sample. The average results over the 4-donor models were reported. The same splits were used for comparative methods.

S3. Evaluation metrics

The performance of our approach and comparative methods was evaluated using Jensen-Shannon Divergence (JSD), Spearman's rank (ρ) correlation coefficient, and PCC. JSD and Spearman's rank (ρ) correlation coefficient are the primary evaluation metrics in our work.

JSD metric serves as a means to quantify the disparity between the predicted and ground truth distributions. It builds upon the Kullback-Leibler divergence and offers enhanced symmetry and bounded characteristics. Its values fall within the interval from 0 to 1. When the JSD reaches 0, it signifies that the predicted probability distributions are in perfect alignment with ground truth distributions. Conversely, a JSD value of 1 indicates that the predicted and ground truth probability distributions could not be more distinct. In general, the closer the JSD value approaches 0, the greater the resemblance between the predicted and ground truth distributions, while a value nearing 1 points to a more pronounced difference. The Spearman's rank correlation coefficient represents a non-parametric statistical measure that gauges the monotonic association between ground truth labels and predicted values. It provides insights into the model's ability to capture rank-order consistency of both variables. The coefficient spans a range from -1 to 1. A value of 1 for the Spearman's rank correlation coefficient signals a precisely positive monotonic relationship between the two variables; a value of -1 denotes a completely

negative monotonic link; and a reading of 0 suggests the absence of any monotonic correlation.

Evaluation metrics were first calculated at the spot-patch level, then averaged across cell types, and finally aggregated over all folds to ensure comprehensive and reliable performance assessment.

In addition, we utilized the bivariate Moran's R index to analyze the spatial autocorrelation manifested between the spatial architectures of different models as well as that in the cell co-localization patterns. This index calculates the spatial weight matrix based on the positional information of the data. By taking into account the proximity of spatial positions, it contrasts the spatial distribution of one set of data with that of another set, thereby gauging the spatial autocorrelation between the two sets of data.

S4. Comparative methods

To verify the effectiveness of our model, we compared it with existing state-of-the-art methods in cell type abundance prediction. We also adapted several gene expression prediction methods for comparison. Brief introductions of the comparative methods are summarized as follows:

- Hist2Cell (Zhao et al. 2024), a vision graph-transformer framework, which predicts fine-grained cell type abundance directly from spot histology images. It is the first method that leverages ST spot patches for fine-grained transcriptional cell types inference.
- ST-Net (He et al. 2020), a deep learning algorithm designed for the prediction of local gene expression from haematoxylin-and-eosin (H&E)-stained histopathology images. It links gene expression with visual features in cell morphology.
- THItoGene (Jia et al. 2024), a neural network framework that utilizes dynamic convolutional and capsule networks to decode spatial gene expression from histological images. This method leverages the spatial arrangement and interaction information for global modeling via the transformer module.
- HisToGene (Pang, Su, and Li 2021), an attention-based model that predicts gene expression from H&E-stained histology images, which leverages positional embedding and considers the spot dependency together with morphological features for gene expression prediction.

S5. Ablation experiments

To further explore the components of our proposed cross-modal representation learning architecture, we conducted ablation experiments focusing on four key aspects: (i) Pathology foundation models, (ii) Cross-modal embedding alignment, (iii) Sensitivity analysis of weights on modalities, (iv) Impact of loss functions, and (v) Impact of latent embedding dimensions.

1. **Pathology foundation models**: We compared the results of applying different pathology foundation models (PFMs) within the morphology-modal representation module. In this ablation experiment, we compared the performance of our CUCA model when focusing on different patch-level PFMs, covering those based on UNI (Chen et al. 2024), CONCH (Lu et al. 2024), Virchow (Vorontsov et al. 2024), Virchow2 (Zimmermann et al. 2024), Prov-GigaPath (Xu et al. 2024), H-optimus-0 (“Releases/Models/h-Optimus/v0 at Main · Bioptimus/Releases,” n.d.), Phikon (Filiot et al. 2023), and PLIP (Huang et al. 2023).
2. **Cross-modal embedding alignment**: We compared the ablation results based solely on morphology with those that integrated morphological embeddings with the molecule modality. This comparison is essential

to understand whether cross-modal embedding alignment is necessary for accurately predicting fine-grained cell type abundances. Specifically, we developed ablated models that utilized pathology foundation models on morphological images. The first model, Morph-LP, was directly trained using the linear probing strategy, while the second model, Morph-Proj, employed the embedding projection head combined with the linear probing. Additionally, we created a cross-modal model, CUCA-fixedFM, which fixed the weight parameters of the PFMs.

3. **Sensitivity analysis of weights on modalities**: We further examined how different modalities contribute to the overall model during cross-modal representation learning. This analysis helped us identify which modalities were most influential in enhancing the model's performance.
4. **Impact of loss functions**: We applied different loss functions to cross-modal representation learning to explore how these loss functions influence the cross-modal representation learning process.
5. **Impact of latent embedding dimensions:** We conducted experiments with latent embedding dimensions of 256, 512, and 1024 while keeping all other hyperparameters fixed. This design aims to evaluate the robustness of the model to latent space dimensionality and allowed us to isolate the effect of the latent space size on cross-modal alignment performance.

S6. Impact of latent embedding dimensions

We investigated the impact of alignment embedding dimensions (256, 512, and 1024) on model performance. Results (Supplementary Fig. S24) showed that CUCA demonstrated consistent performance across all tested dimensions, with stable Jensen-Shannon Divergence (JSD) and Spearman's ρ values. The Friedman test confirmed no statistically significant differences ($p > 0.05$) between dimensional configurations. These results demonstrate the dimensional robustness of our model architecture.

References

Andersson, Alma, Ludvig Larsson, Linnea Stenbeck, Fredrik Salmén, Anna Ehinger, Sunny Z. Wu, Ghamdan Al-Eryani, et al. 2021. “Spatial Deconvolution of HER2-Positive Breast Cancer Delineates Tumor-Associated Cell Type Interactions.” *Nature Communications* 12 (1): 6012. https://doi.org/10.1038/s41467-021-26271-2.

Chen, Richard J., Tong Ding, Ming Y. Lu, Drew F. K. Williamson, Guillaume Jaume, Andrew H. Song, Bowen Chen, et al. 2024. “Towards a General-Purpose Foundation Model for Computational Pathology.” *Nature Medicine* 30 (3): 850–62. https://doi.org/10.1038/s41591-024-02857-3.

Filiot, Alexandre, Ridouane Ghermi, Antoine Olivier, Paul Jacob, Lucas Fidon, Alice Mac Kain, Charlie Saillard, and Jean-Baptiste Schiratti. 2023. “Scaling Self-Supervised Learning for Histopathology with Masked Image Modeling.” https://doi.org/10.1101/2023.07.21.23292757.

He, Bryan, Ludvig Bergenstråhle, Linnea Stenbeck, Abubakar Abid, Alma Andersson, Åke Borg, Jonas Maaskola, Joakim Lundeberg, and James Zou. 2020. “Integrating Spatial Gene Expression and Breast Tumour Morphology via Deep Learning.” *Nature Biomedical Engineering* 4 (8): 827–34. https://doi.org/10.1038/s41551-020-0578-x.

Huang, Zhi, Federico Bianchi, Mert Yuksekgonul, Thomas J. Montine, and James Zou. 2023. “A Visual–Language Foundation Model for Pathology Image Analysis Using Medical Twitter.” *Nature Medicine* 29 (9): 2307–16. https://doi.org/10.1038/s41591-023-02504-3.

Jia, Yuran, Junliang Liu, Li Chen, Tianyi Zhao, and Yadong Wang. 2024. “THItoGene: A Deep Learning Method for Predicting Spatial Transcriptomics from Histological Images.” *Briefings in Bioinformatics* 25 (1): bbad464. https://doi.org/10.1093/bib/bbad464.

Kumar, Tapsi, Kevin Nee, Runmin Wei, Siyuan He, Quy H. Nguyen, Shanshan Bai, Kerrigan Blake, et al. 2023. “A Spatially Resolved Single-Cell Genomic Atlas of the Adult Human Breast.” *Nature*, June, 1–11. https://doi.org/10.1038/s41586-023-06252-9.

Lu, Ming Y., Bowen Chen, Drew F. K. Williamson, Richard J. Chen, Ivy Liang, Tong Ding, Guillaume Jaume, et al. 2024. “A Visual-Language Foundation Model for Computational Pathology.” *Nature Medicine* 30 (3): 863–74. https://doi.org/10.1038/s41591-024-02856-4.

Madissoon, Elo, Amanda J. Oliver, Vitalii Kleshchevnikov, Anna Wilbrey-Clark, Krzysztof Polanski, Nathan Richoz, Ana Ribeiro Orsi, et al. 2023. “A Spatially Resolved Atlas of the Human Lung Characterizes a Gland-Associated Immune Niche.” *Nature Genetics* 55 (1): 66–77. https://doi.org/10.1038/s41588-022-01243-4.

Pang, Minxing, Kenong Su, and Mingyao Li. 2021. “Leveraging Information in Spatial Transcriptomics to Predict Super-Resolution Gene Expression from Histology Images in Tumors.” bioRxiv. https://doi.org/10.1101/2021.11.28.470212.

“Releases/Models/h-Optimus/v0 at Main · Bioptimus/Releases.” n.d. GitHub. Accessed September 25, 2024. https://github.com/bioptimus/releases/tree/main/models/h-optimus/v0.

Smith, Leslie N., and Nicholay Topin. 2019. “Super-Convergence: Very Fast Training of Neural Networks Using Large Learning Rates.” In *Artificial Intelligence and Machine Learning for Multi-Domain Operations Applications*, 11006:369–86. SPIE. https://doi.org/10.1117/12.2520589.

Ståhl, Patrik L., Fredrik Salmén, Sanja Vickovic, Anna Lundmark, José Fernández Navarro, Jens Magnusson, Stefania Giacomello, et al. 2016. “Visualization and Analysis of Gene Expression in Tissue Sections by Spatial Transcriptomics.” *Science* 353 (6294): 78–82. https://doi.org/10.1126/science.aaf2403.

Vorontsov, Eugene, Alican Bozkurt, Adam Casson, George Shaikovski, Michal Zelechowski, Kristen Severson,

Eric Zimmermann, et al. 2024. “A Foundation Model for Clinical-Grade Computational Pathology and Rare Cancers Detection.” *Nature Medicine*, July, 1–12. https://doi.org/10.1038/s41591-024-03141-0.

Xu, Hanwen, Naoto Usuyama, Jaspreet Bagga, Sheng Zhang, Rajesh Rao, Tristan Naumann, Cliff Wong, et al. 2024. “A Whole-Slide Foundation Model for Digital Pathology from Real-World Data.” *Nature*, May, 1–8. https://doi.org/10.1038/s41586-024-07441-w.

Zhao, Weiqin, Zhuo Liang, Xianjie Huang, Yuanhua Huang, and Lequan Yu. 2024. “Hist2Cell: Deciphering Fine-Grained Cellular Architectures from Histology Images.” bioRxiv. https://doi.org/10.1101/2024.02.17.580852.

Zimmermann, Eric, Eugene Vorontsov, Julian Viret, Adam Casson, Michal Zelechowski, George Shaikovski, Neil Tenenholtz, et al. 2024. “Virchow 2: Scaling Self-Supervised Mixed Magnification Models in Pathology.” arXiv. http://arxiv.org/abs/2408.00738.

Supplementary Tables

Table S1. Comparison results on our model, CUCA, against comparative methods in terms of **Jensen-Shannon Divergence (JSD)** evaluation metric at **split (fold) -level**. The results were evaluated on humanlung, her2st, and stnet datasets. Mean and standard deviation are reported, and the best results are marked in **bold**. (The closer the JSD value approaches 0, the better the model performance evaluation)

**Split (Fold) -level**	humanlung	her2st	stnet
CUCA (ours)	**0.166±0.023**	**0.121±0.024**	**0.103±0.017**
Hist2Cell	0.205±0.024	0.123±0.026	0.123±0.018
LinearProbing	0.227±0.045	0.127±0.034	0.117±0.018
HisToGene	0.287±0.037	0.157±0.030	0.155±0.022
THItoGene	0.304±0.035	0.194±0.033	0.148±0.021
ST-Net	0.220±0.027	0.130±0.032	0.128±0.018

Table S2. Comparison results on our model, CUCA, against comparative methods in terms of **Spearman's rank correlation coefficient** evaluation metric at **split (fold) -level**. The results were evaluated on humanlung, her2st, and stnet datasets. Mean and standard deviation are reported, and the best results are marked in **bold**. (The closer the Spearman's rank correlation coefficient approaches 1, the better the model performance)

**Split (Fold) -level**	humanlung	her2st	stnet
CUCA (ours)	**0.376±0.080**	**0.376±0.088**	**0.306±0.039**
Hist2Cell	0.307±0.095	0.375±0.105	0.251±0.031
LinearProbing	0.306±0.037	0.331±0.112	0.252±0.062
HisToGene	0.093±0.096	0.225±0.148	0.138±0.026
THItoGene	0.019±0.034	-0.025±0.071	-0.031±0.017
ST-Net	0.260±0.102	0.346±0.105	0.207±0.061

Table S3. Comparison results on our model, CUCA, against comparative methods in terms of **Pearson correlation coefficient** evaluation metric at **split (fold) -level**. The results were evaluated on humanlung, her2st, and stnet datasets. Mean and standard deviation are reported, and the best results are marked in **bold**. (The closer the Pearson correlation coefficient approaches 1, the better the model performance evaluation)

**Split (Fold) -level**	humanlung	her2st	stnet
CUCA (ours)	**0.375±0.057**	**0.353±0.076**	**0.273±0.018**
Hist2Cell	0.305±0.092	0.334±0.058	0.235±0.020
LinearProbing	0.324±0.059	0.324±0.075	0.237±0.032
HisToGene	0.085±0.074	0.210±0.115	0.212±0.036
THItoGene	0.038±0.041	0.085±0.055	0.139±0.075
ST-Net	0.275±0.099	0.299±0.058	0.201±0.037

Table S4. Comparison results on our model, CUCA, against comparative methods in terms of **Jensen-Shannon Divergence (JSD)** evaluation metric at **sample (slice) -level**. The results were evaluated on humanlung, her2st, and stnet datasets. Mean and standard deviation are reported, and the best results are marked in **bold**. (The closer the JSD value approaches 0, the better the model performance evaluation)

**Sample (Slice) -level**	humanlung	her2st	stnet
CUCA (ours)	**0.391±0.029**	**0.301±0.028**	**0.267±0.029**
Hist2Cell	0.438±0.028	0.303±0.039	0.288±0.034
LinearProbing	0.464±0.048	0.318±0.050	0.290±0.035
HisToGene	0.523±0.039	0.360±0.049	0.347±0.036
THItoGene	0.530±0.031	0.391±0.063	0.309±0.045
ST-Net	0.455±0.027	0.312±0.039	0.296±0.030

Table S5. Comparison results on our model, CUCA, against comparative methods in terms of **Spearman's rank correlation coefficient** evaluation metric at **sample (slice) -level**. The results were evaluated on humanlung, her2st, and stnet datasets. Mean and standard deviation are reported, and the best results are marked in **bold**. (The closer the Spearman's rank correlation coefficient approaches 1, the better the model performance)

**Sample (Slice) -level**	humanlung	her2st	stnet
CUCA (ours)	**0.328±0.075**	0.372±0.180	**0.267±0.183**
Hist2Cell	0.250±0.058	**0.380±0.193**	0.259±0.172
LinearProbing	0.257±0.056	0.324±0.166	0.238±0.160
HisToGene	0.036±0.018	0.245±0.148	0.127±0.150
THItoGene	0.005±0.052	0.048±0.112	0.034±0.136
ST-Net	0.202±0.041	0.355±0.182	0.218±0.171

Table S6. Comparison results on our model, CUCA, against comparative methods in terms of **Pearson correlation coefficient** evaluation metric at **sample (slice) -level**. The results were evaluated on humanlung, her2st, and stnet datasets. Mean and standard deviation are reported, and the best results are marked in **bold**. (The closer the Pearson correlation coefficient approaches 1, the better the model performance evaluation)

**Sample (Slice) -level**	humanlung	her2st	stnet
CUCA (ours)	**0.340±0.081**	**0.397±0.199**	0.246±0.169
Hist2Cell	0.271±0.063	0.383±0.190	0.239±0.161
LinearProbing	0.291±0.041	0.366±0.170	0.229±0.151
HisToGene	0.039±0.024	0.239±0.150	**0.251±0.137**
THItoGene	0.001±0.029	0.065±0.078	0.158±0.097
ST-Net	0.243±0.064	0.360±0.194	0.205±0.162

Supplementary Figures

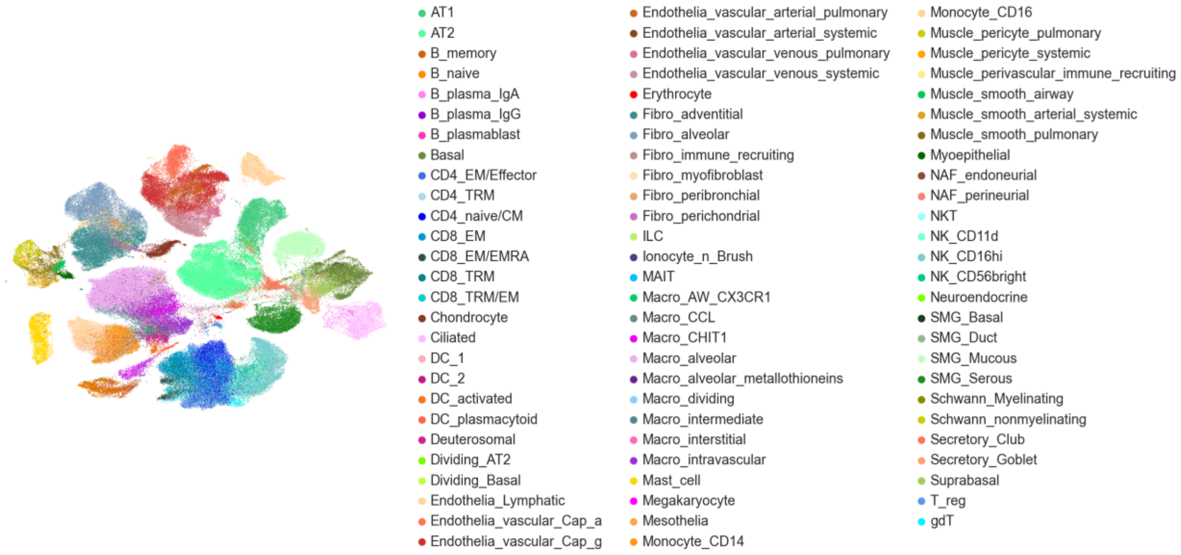

Figure S1. UMAP visualization of 80 fine-grained cell types on the single-cell reference dataset for humanlung dataset. The names of 80 fine-grained cell types are also listed.

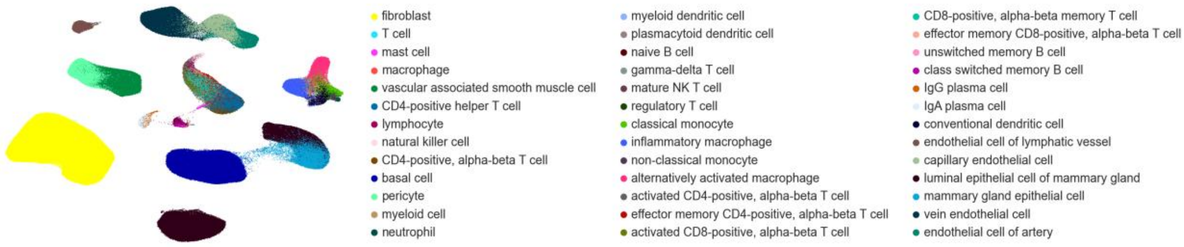

Figure S2. UMAP visualization of 39 fine-grained cell types on the single-cell reference dataset for her2st and stnet breast cancer dataset. The names of 39 fine-grained cell types are also listed.

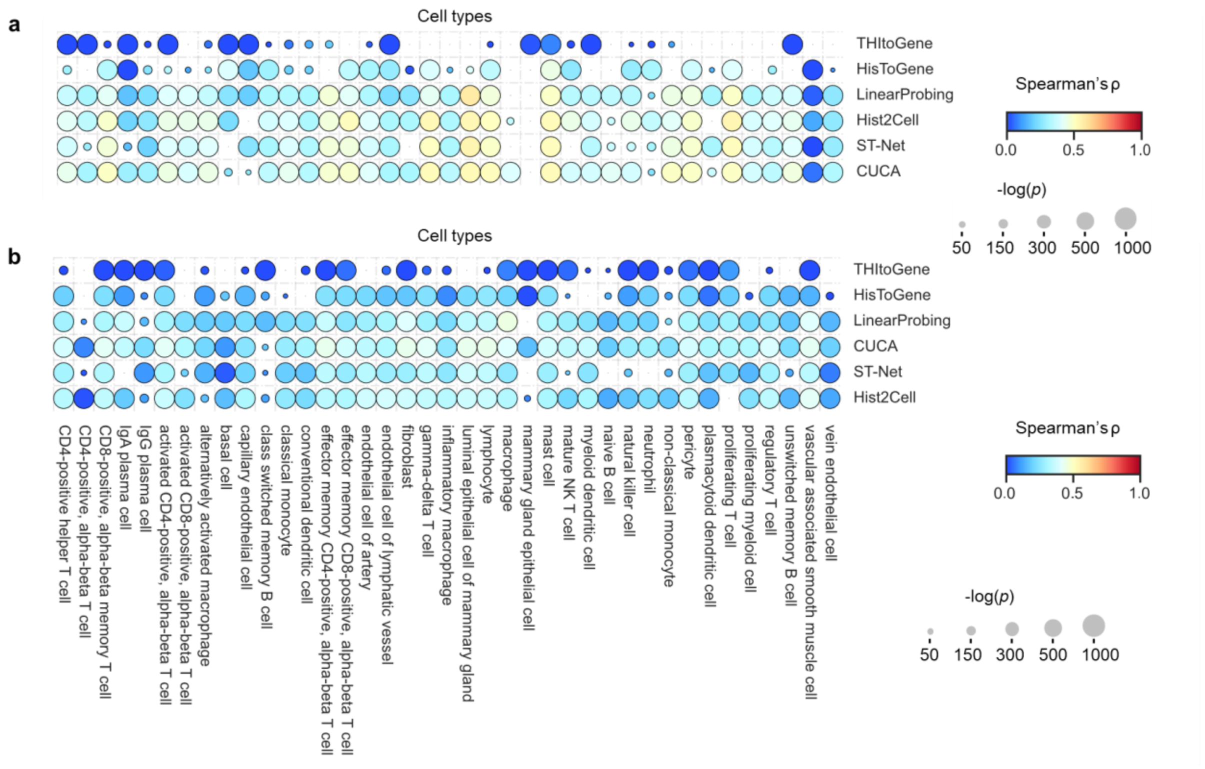

Figure S3. Dot plot comparing the Spearman's ρ correlation performance of 39 fine-grained cell types in the (a) **her2st** and (b) **stnet** dataset, with color indicating Spearman's ρ and size representing the negative logarithm of the p-value (two-sided t-test approximation).

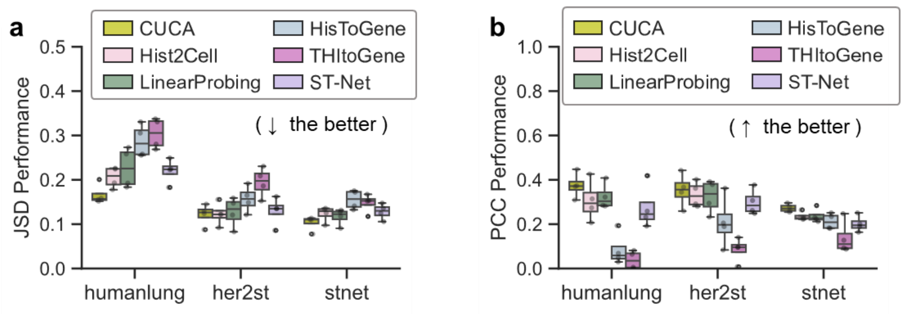

Figure S4. Split-level averaged (a) Jensen-Shannon Divergence (JSD) and (b) Pearson correlation coefficient (PCC) performance on the humanlung, her2st, and stnet datasets.

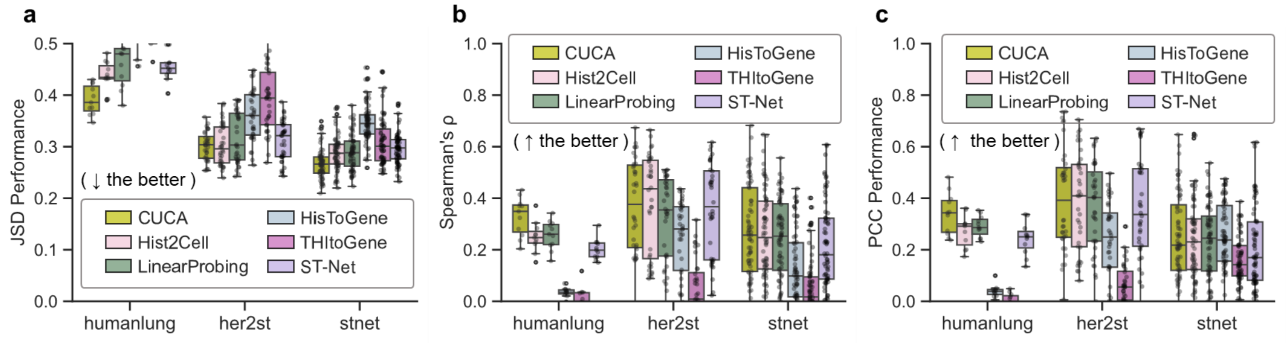

Figure S5. Slice-level averaged (a) Jensen-Shannon Divergence (JSD), (b) Spearman's ρ correlation coefficient, and (c) Pearson correlation coefficient (PCC) performance on the humanlung, her2st, and stnet datasets.

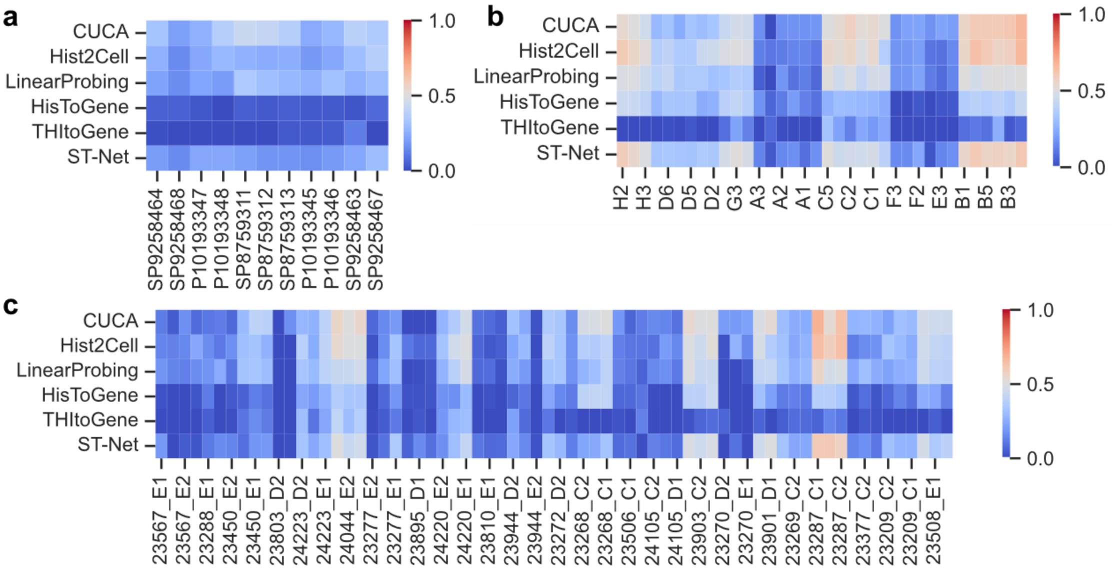

Figure S6. Heatmaps comparing Spearman's ρ performance of all samples in the (a) **humanlung**, (b) **her2st**, and (c) **stnet** datasets, respectively. Color bar represents Spearman's ρ performance levels, ranging from 0 (blue) to 1 (red).

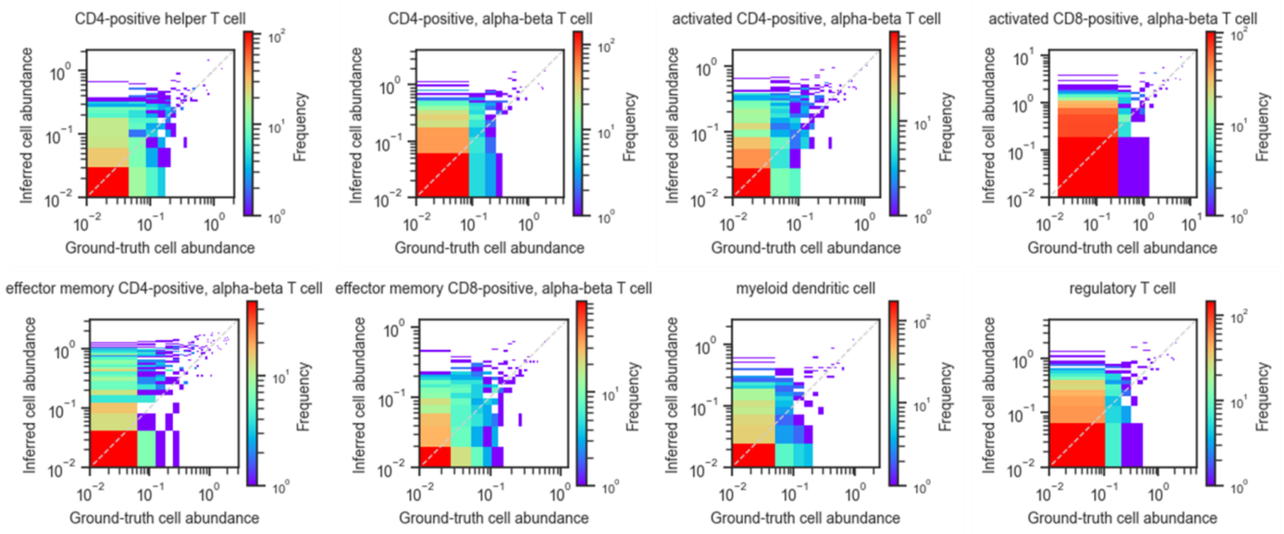

Evaluation metrics	CD4-positive helper T cell	CD4-positive, alpha-beta T cell	activated CD4-positive, alpha-beta T cell	activated CD8-positive, alpha-beta T cell	effector memory CD4-positive, alpha-beta T cell	effector memory CD8-positive, alpha-beta T cell	myeloid dendritic cell	regulatory T cell
**JSD (↓)**	0.110	0.143	0.117	0.138	0.134	0.111	0.181	0.141
**Spearman's ρ (↑)**	0.578	0.517	0.579	0.405	0.610	0.537	0.380	0.447
**PCC (↑)**	0.623	0.642	0.621	0.708	0.614	0.627	0.604	0.653

Figure S7. Log-scale 2D histograms of predicted vs. ground truth abundances for key cell types in CUCA, with the first diagonal (y=x) reference line and color-coded 2D histogram counts. The cases are derived from breast cancer samples (the **her2st** dataset). Quantitative evaluation results including JSD (Jensen-Shannon Divergence), Spearman's ρ, and PCC (Pearson correlation coefficient) for each cell type are listed.

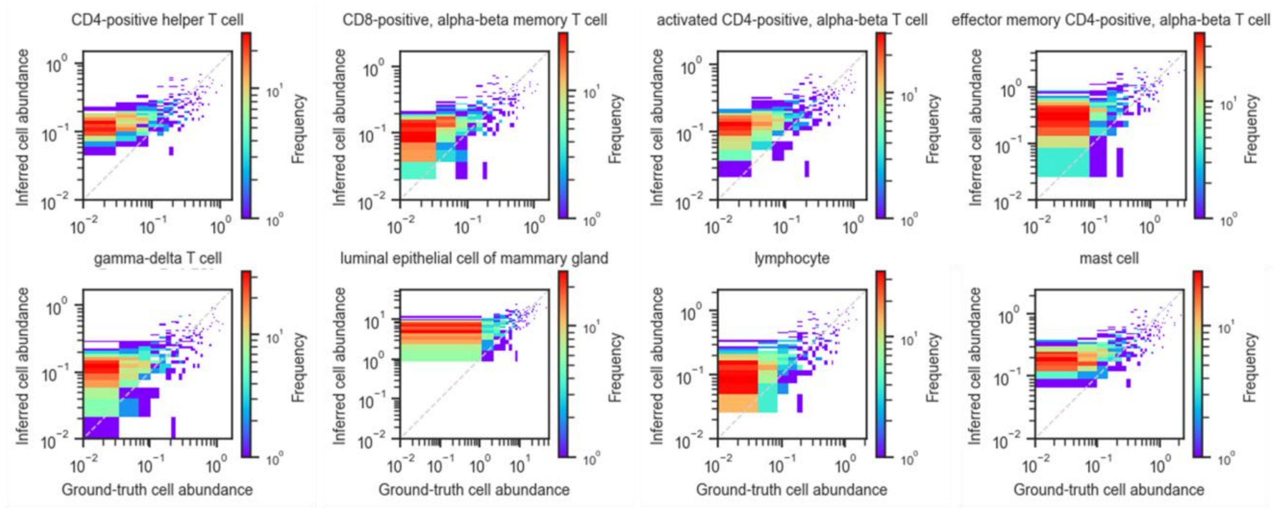

Evaluation metrics	CD4-positive helper T cell	CD8-positive, alpha-beta memory T cell	activated CD4-positive, alpha-beta T cell	effector memory CD4-positive, alpha-beta T cell	gamma-delta T cell	luminal epithelial cell of mammary gland	lymphocyte	mast cell
**JSD (↓)**	0.083	0.086	0.085	0.118	0.082	0.096	0.087	0.077
**Spearman's ρ (↑)**	0.666	0.699	0.664	0.662	0.679	0.662	0.709	0.678
**PCC (↑)**	0.751	0.765	0.754	0.775	0.753	0.788	0.774	0.770

Figure S8. Log-scale 2D histograms of predicted vs. ground truth abundances for key cell types in CUCA, with the first diagonal (y=x) reference line and color-coded 2D histogram counts. The cases are derived from breast cancer samples (the **stnet** dataset). Quantitative evaluation results including JSD (Jensen-Shannon Divergence), Spearman's ρ, and PCC (Pearson correlation coefficient) for each cell type are listed.

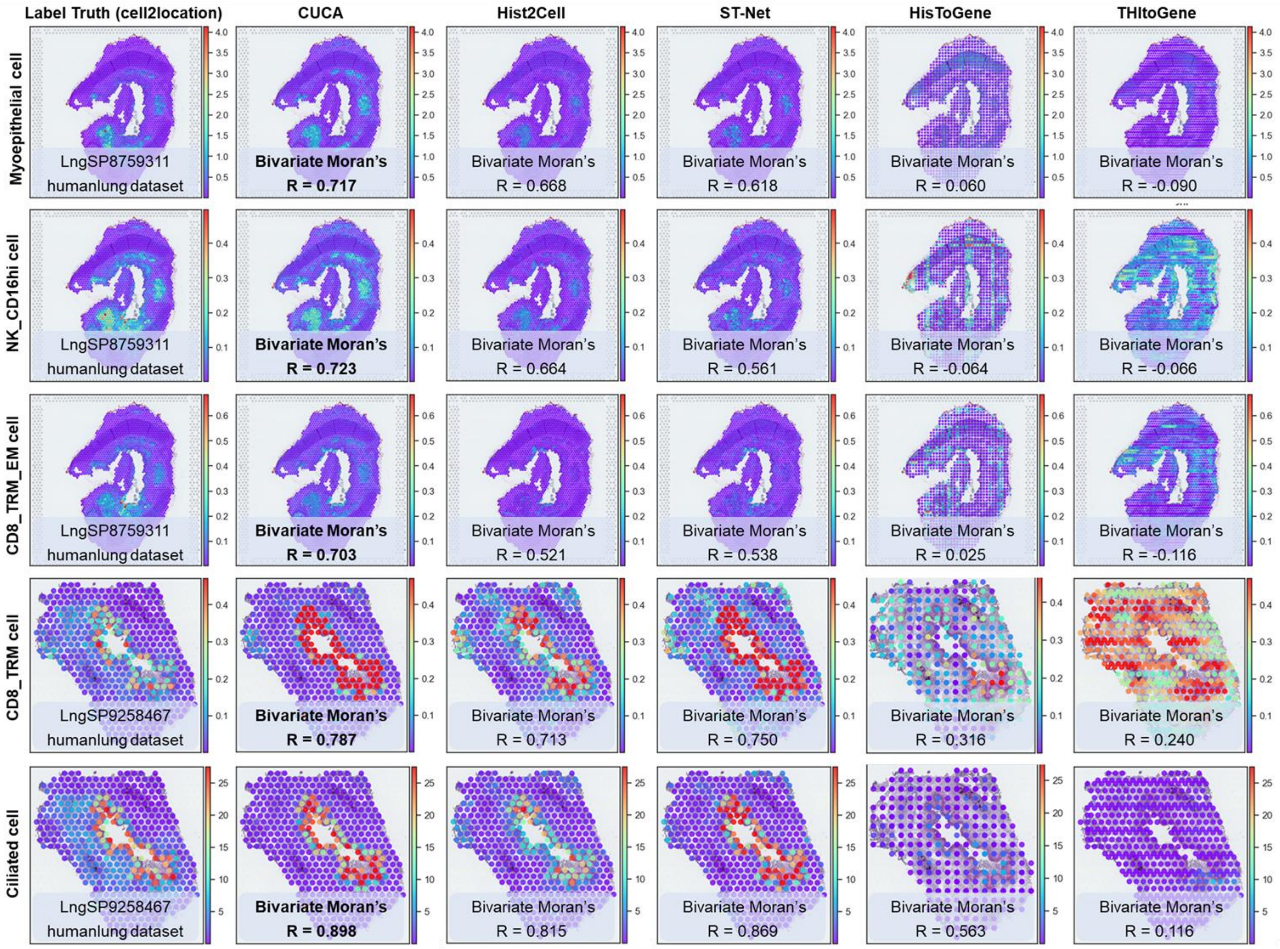

Figure S9. Spatial distribution of predicted cell abundances for key fine-grained cell types, with color scales aligned to that of label truth for each cell type. Bivariate Moran's R index, measuring spatial correlation, is displayed for each plot. Color bar represents cell abundance levels. All samples are from the **humanlung** dataset.

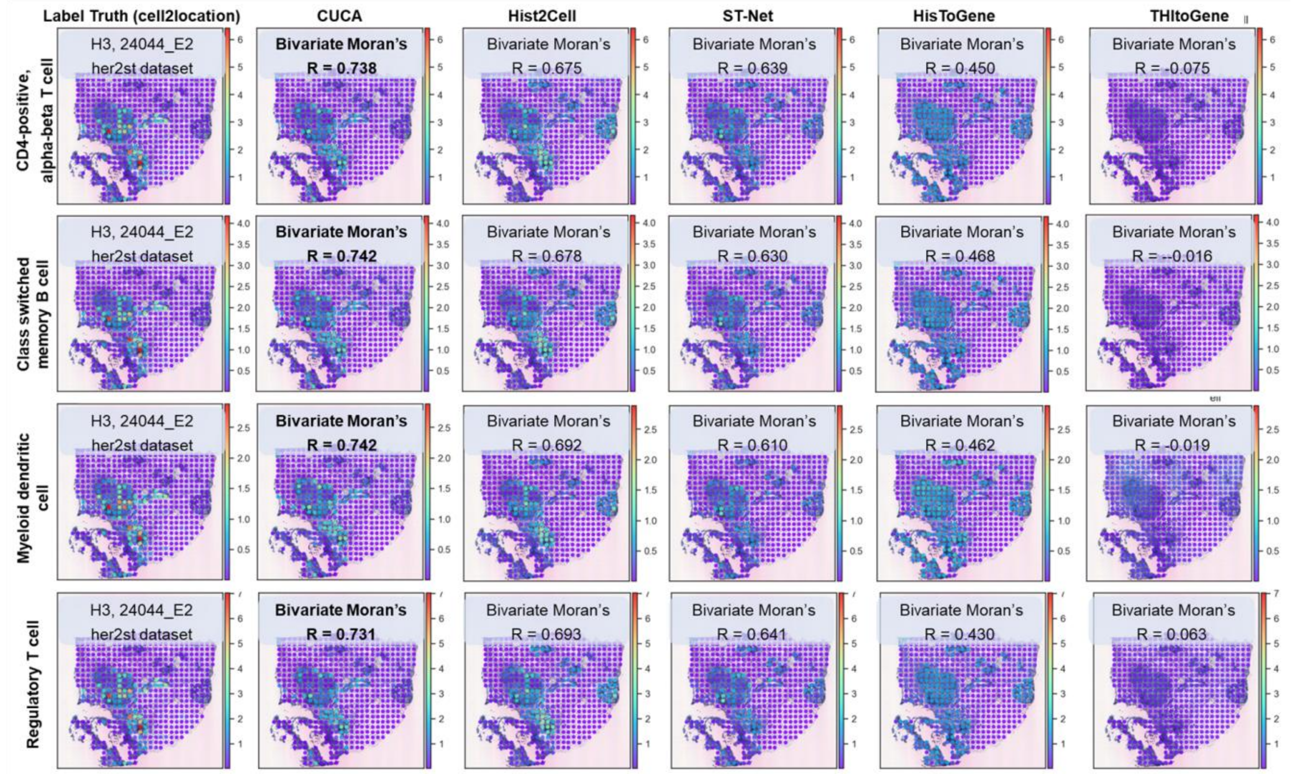

Figure S10. Spatial distribution of predicted cell abundances for key fine-grained cell types, with color scales aligned to that of label truth for each cell type. Bivariate Moran's R index, measuring spatial correlation, is displayed for each plot. Color bar represents cell abundance levels. All samples are from the **her2st** dataset.

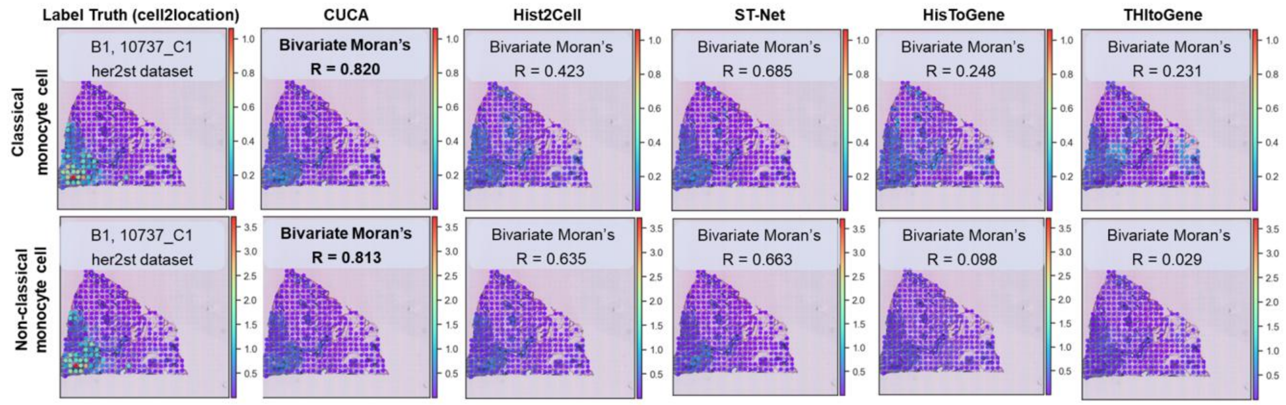

Figure S11. Spatial distribution of predicted cell abundances for key fine-grained cell types, with color scales aligned to that of label truth for each cell type. Bivariate Moran's R index, measuring spatial correlation, is displayed for each plot. Color bar represents cell abundance levels. All samples are from the **stnet** dataset.

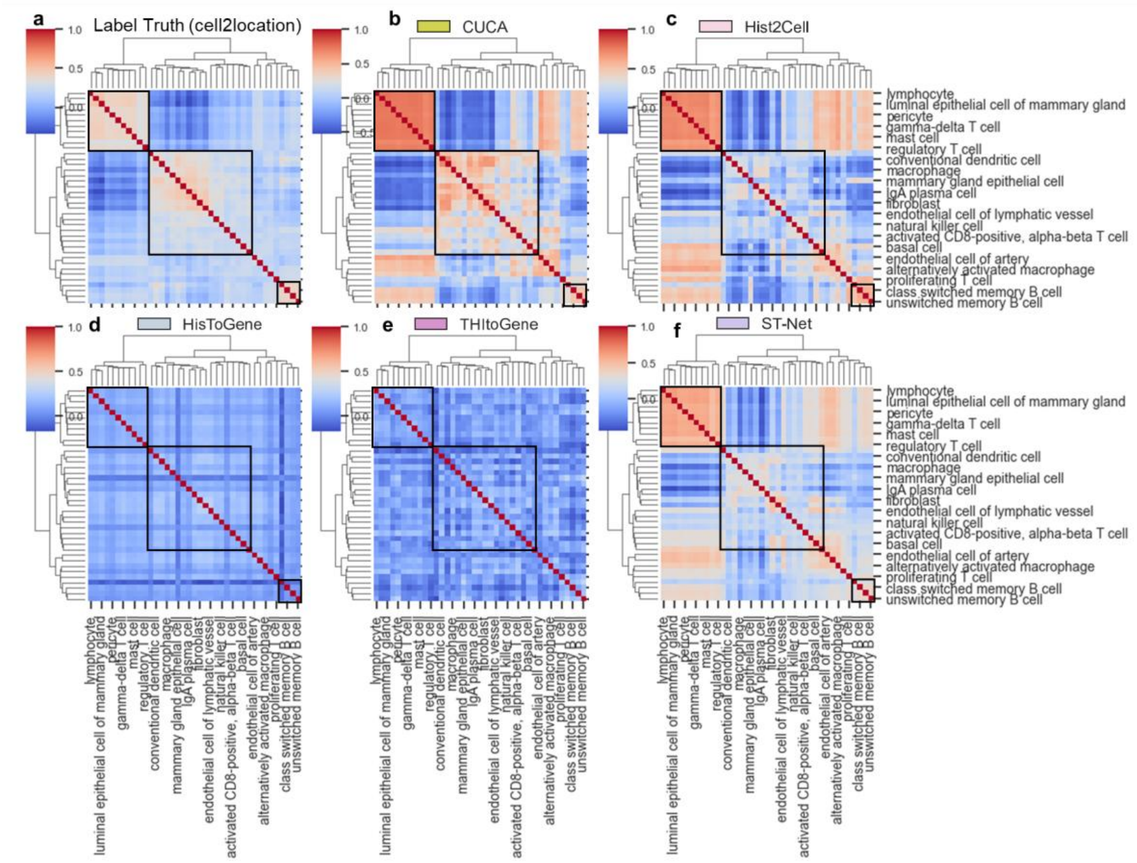

Figure S12. Hierarchically clustered heatmaps of spatial correlations (bivariate Moran's R) between cell type pairs in **stnet** dataset: (a) ground truth, (b) CUCA, and (c-f) comparative methods (key patterns boxed). Color bar represents bivariate Moran's R levels between cell type pairs.

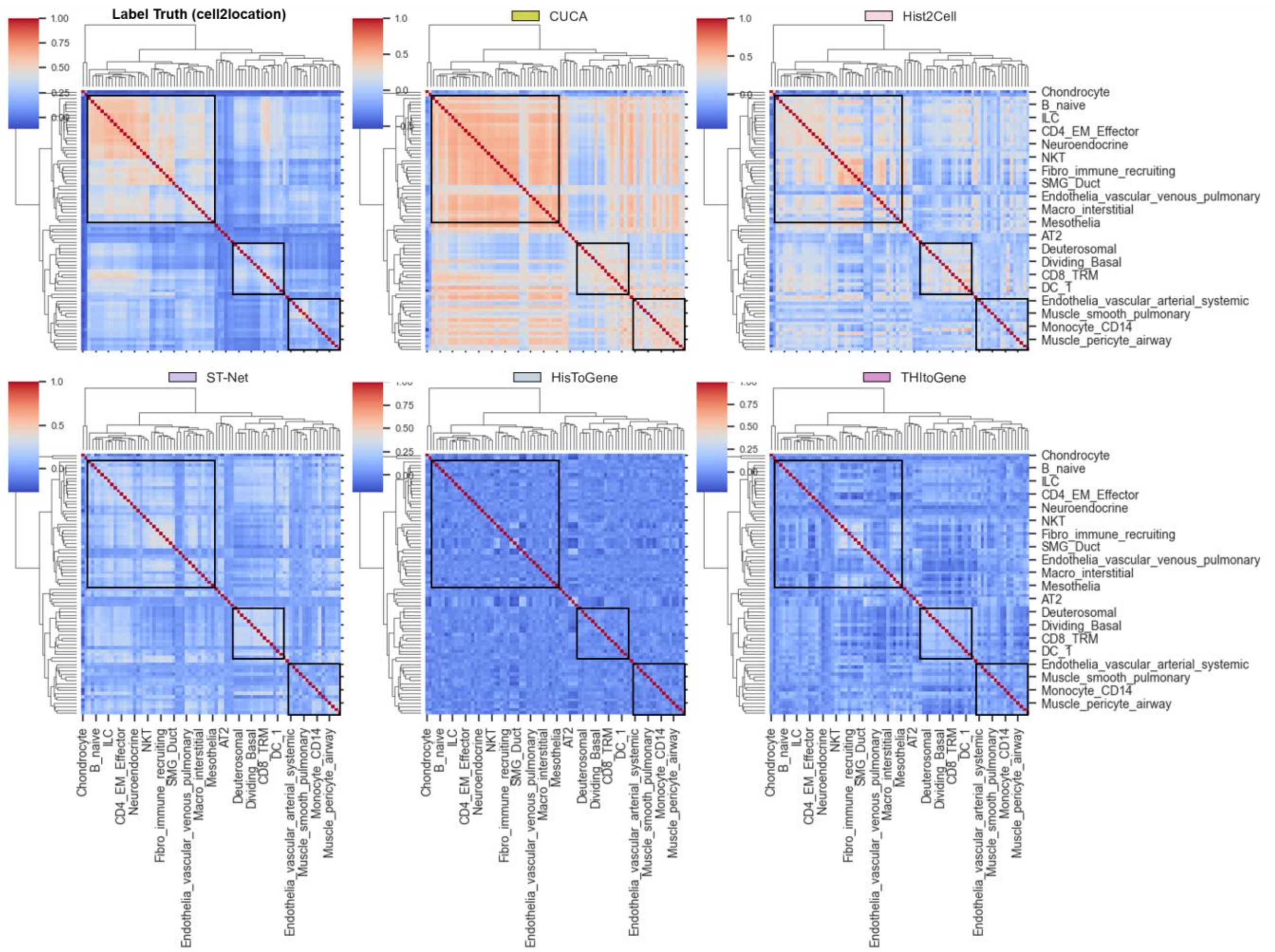

Figure S13. Hierarchically clustered heatmaps of spatial correlations (bivariate Moran's R) between cell type pairs in **humanlung** dataset: (a) ground truth, (b) CUCA, and (c-f) comparative methods (key patterns boxed). Color bar represents bivariate Moran's R levels between cell type pairs.

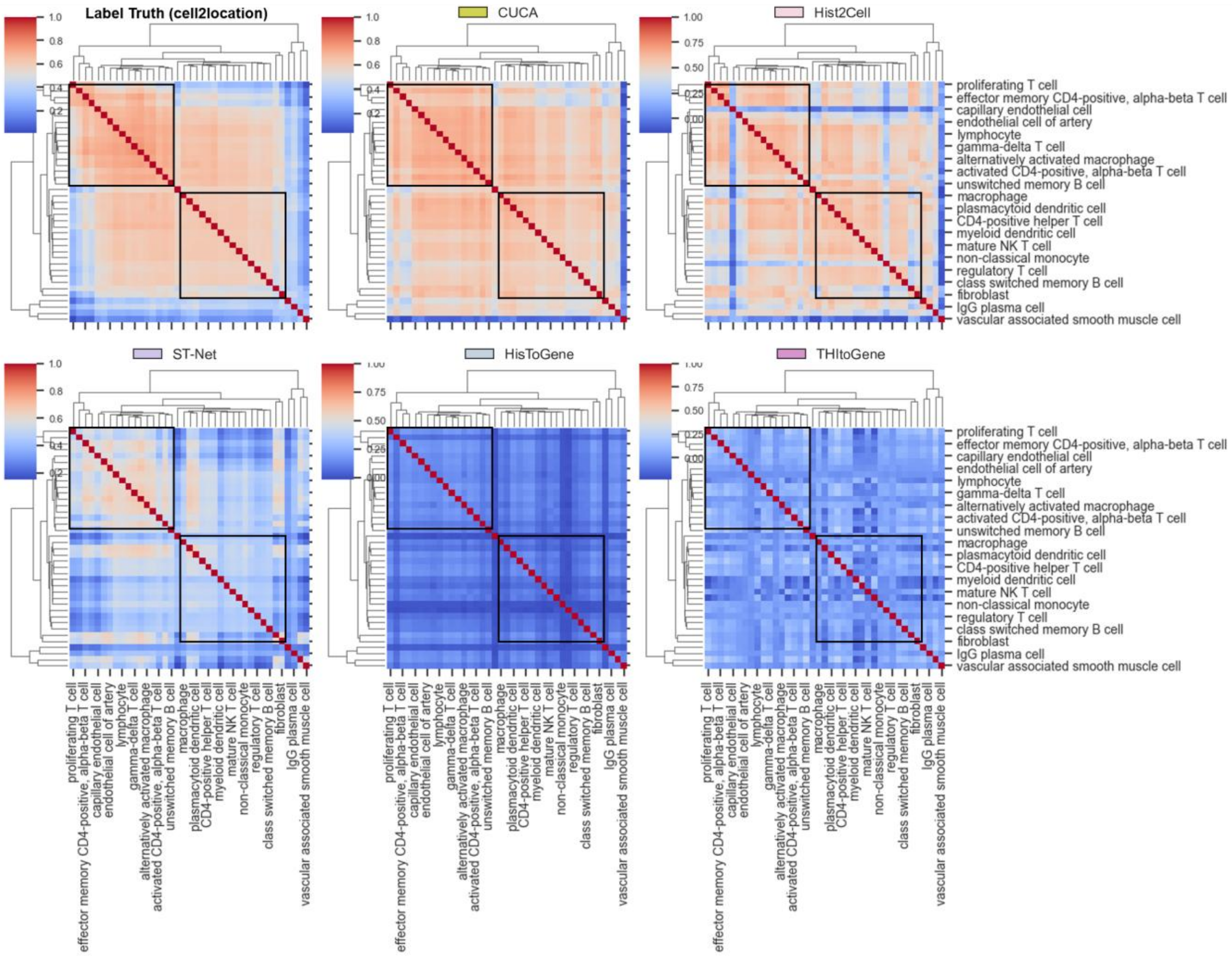

Figure S14. Hierarchically clustered heatmaps of spatial correlations (bivariate Moran's R) between cell type pairs in **her2st** dataset: (a) ground truth, (b) CUCA, and (c-f) comparative methods (key patterns boxed). Color bar represents bivariate Moran's R levels between cell type pairs.

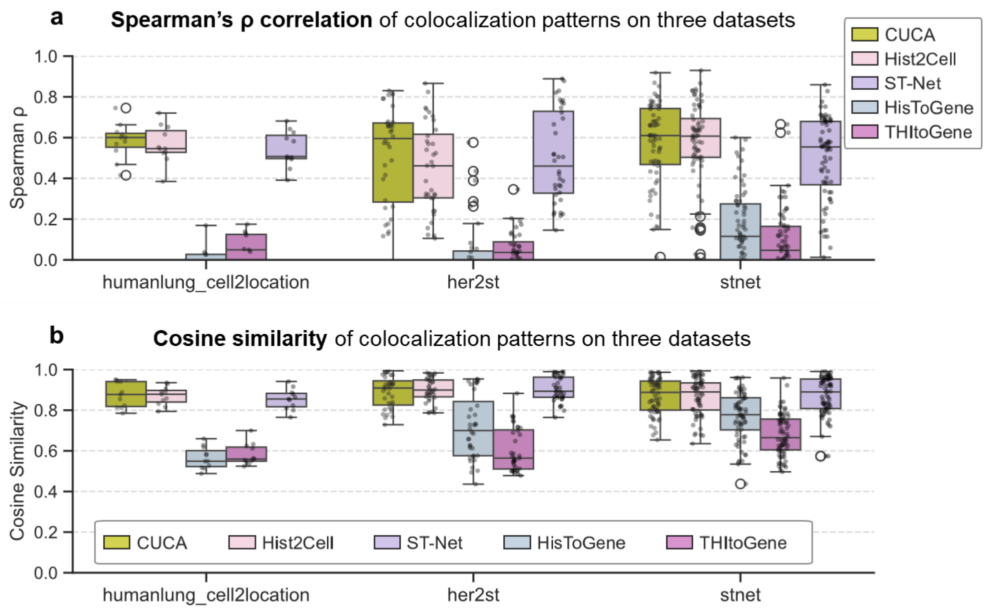

Figure S15. Results of (a) Spearman's ρ correlation performance and (b) Cosine similarity comparing predicted versus ground truth global co-localization patterns across three datasets (humanlung, her2st, and stnet).

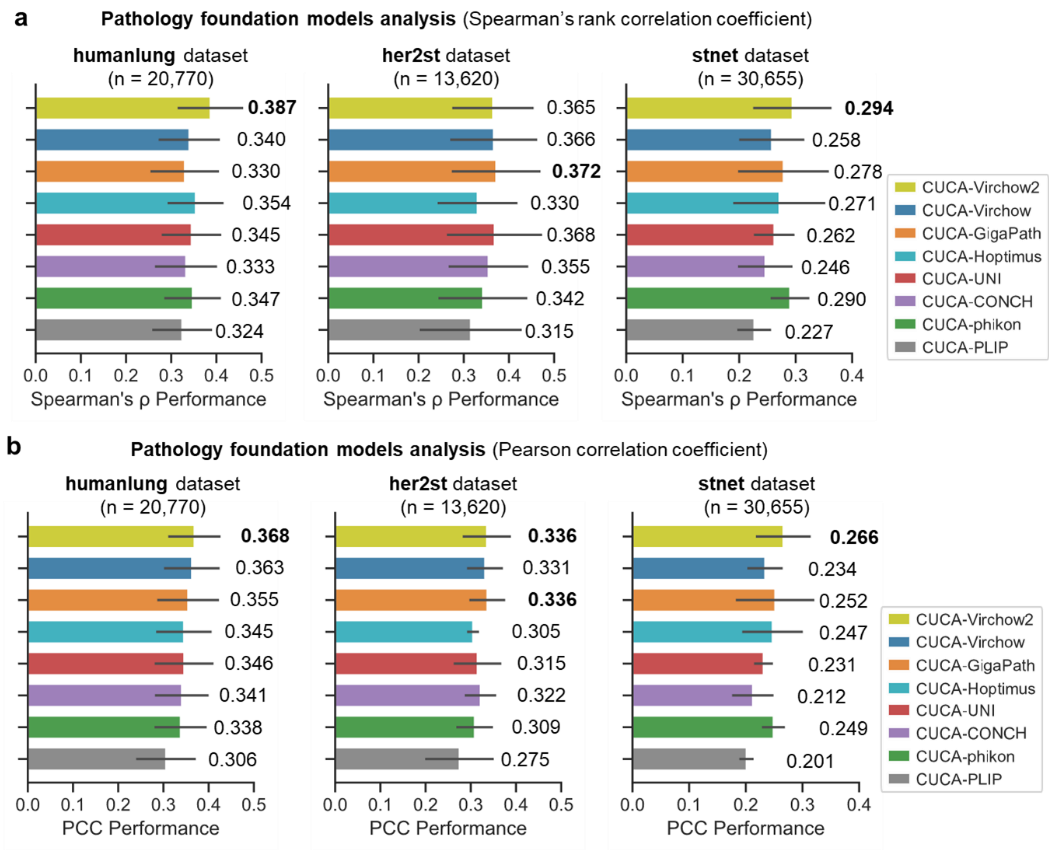

Figure S16. Evaluation results of Ablation study of **pathology foundation models** in terms of (a) Spearman' s ρ correlation and (b) Pearson correlation coefficient (PCC) metrics across three datasets (humanlung, her2st, and stnet). Mean values are shown on the plot with standard deviation indicated by error lines. Best performance is highlighted in **bold**.

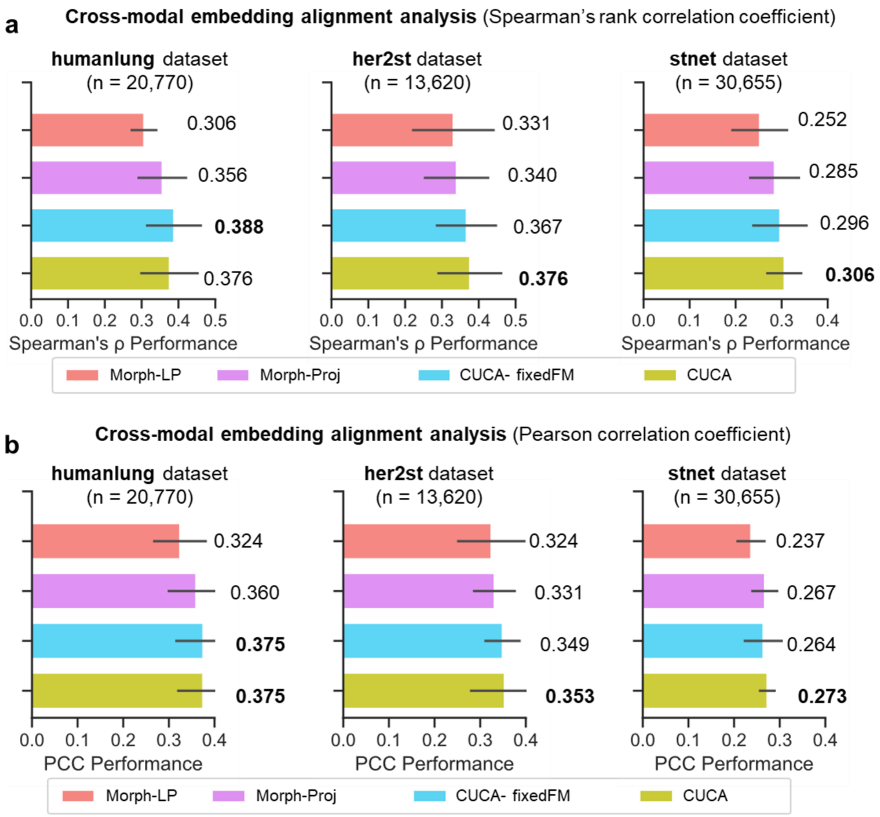

Figure S17. Evaluation results of Ablation study of **Cross-modal embedding alignment** in terms of (a) Spearman's ρ correlation and (b) Pearson correlation coefficient (PCC) metrics across three datasets (humanlung, her2st, and stnet). Mean values are shown on the plot with standard deviation indicated by error lines. Best performance is highlighted in **bold**.

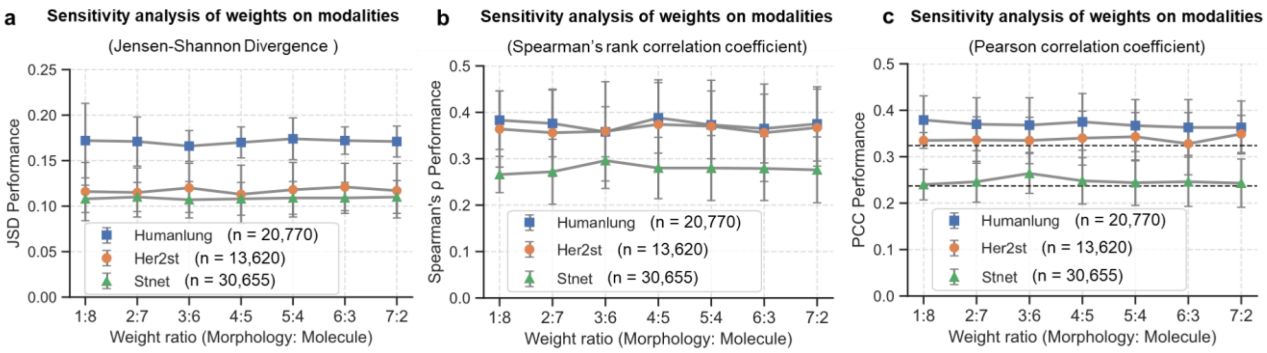

Figure S18. Evaluation results of Ablation study of **Sensitivity analysis of weights on modalities** in terms of (a) Jensen-Shannon Divergence (JSD), (b) Spearman' s ρ correlation, and (c) Pearson correlation coefficient (PCC) metrics across three datasets (humanlung, her2st, and stnet). Mean values are plotted with standard deviation indicated by error lines.

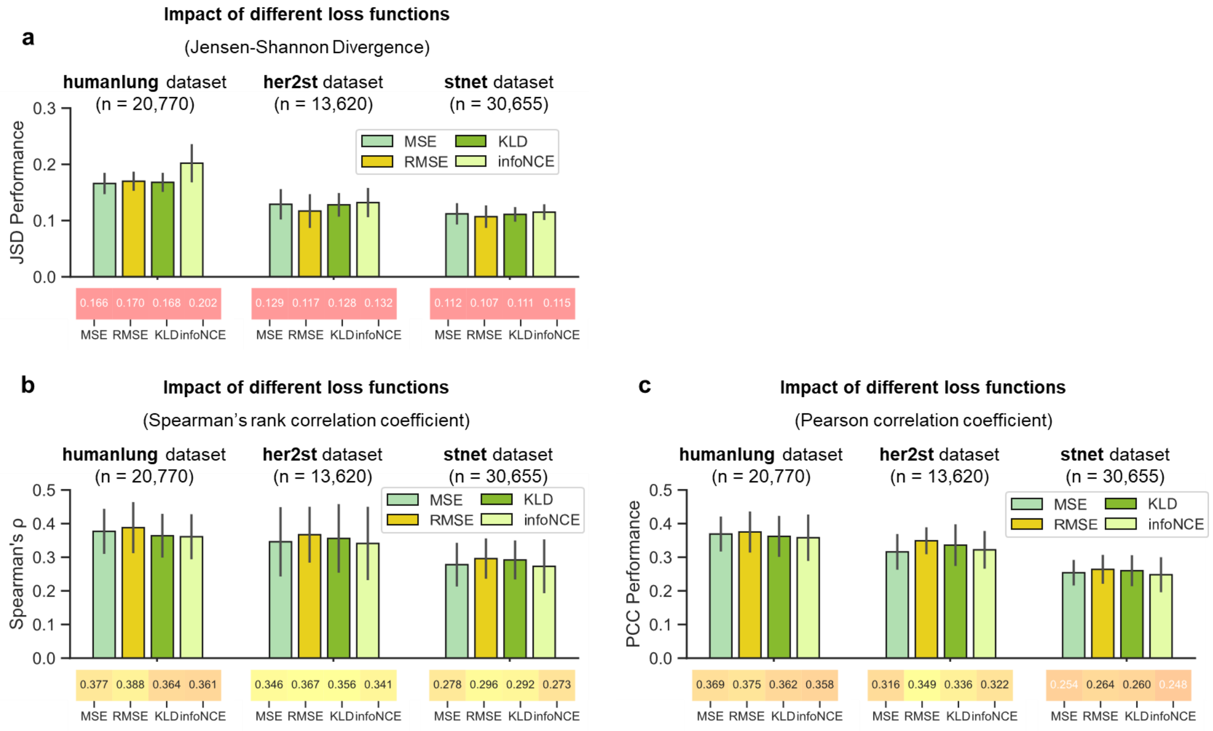

Figure S19. Evaluation results of Ablation study of **Impact of different loss functions** in terms of (a) Jensen-Shannon Divergence (JSD), (b) Spearman' s ρ correlation, and (c) Pearson correlation coefficient (PCC) metrics across three datasets (humanlung, her2st, and stnet). Mean values are displayed at the bottom of the plots, with standard deviation indicated by error lines.

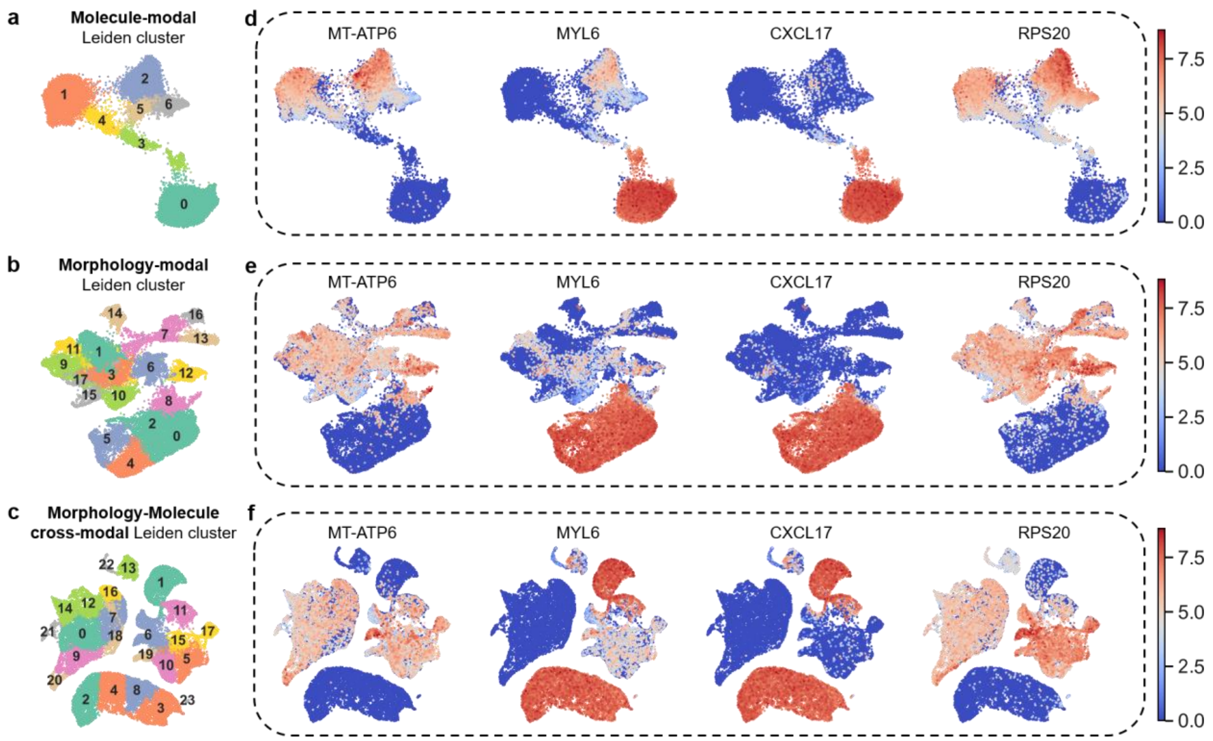

Figure S20. UMAP visualization of Leiden clustering on the embeddings of (a) molecule-only, (b) morphology-only, and (c) morphology-molecule cross-modal data across spots in **humanlung** ST samples. (d-f) UMAP appearance of key highly expressed genes, including MT-ATP6, MYL6, CXCL17, and RPS20, on (d) molecule-only, (e) morphology-only, and (f) morphology-molecule cross-modal embeddings, respectively. The color indicates the expression levels of specific genes.

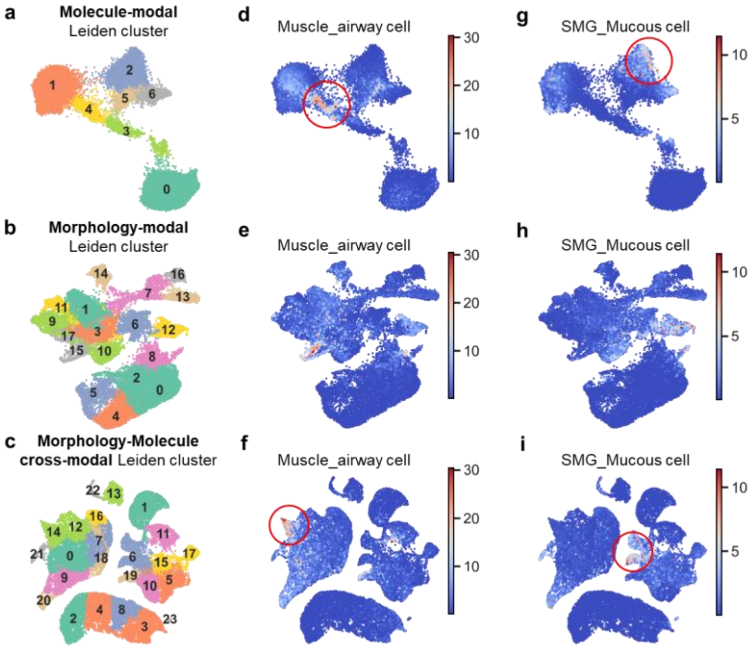

Figure S21. UMAP visualizations of **humanlung** ST sample embeddings: (a-c) Cluster distributions for (a) molecular, (b) morphological, and (c) cross-modal embeddings (colors=cluster IDs). (d-i) Cell type-specific patterns for (d-f) Muscle_airway and (g-i) SMG_Mucous cells, with color intensity reflecting cell abundance.

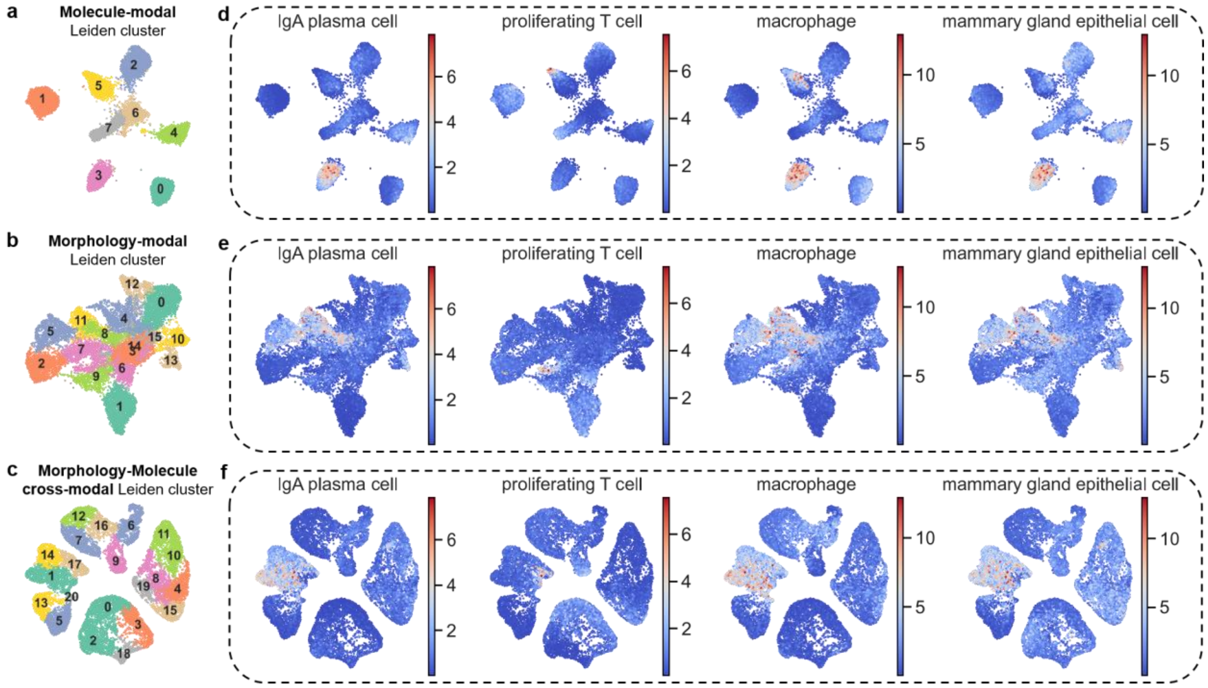

Figure S22. UMAP visualization of Leiden clustering on the embeddings of (a) molecule-only, (b) morphology-only, and (c) morphology-molecule cross-modal data across spots in **her2st** ST samples. (d-f) UMAP appearance of key fine-grained cell types, including IgA plasma cell, proliferating T cell, macrophage cell, and mammary gland epithelial cells, on (d) molecule-only, (e) morphology-only, and (f) morphology-molecule cross-modal embeddings, respectively. The color indicates the abundance values of specific cell types.

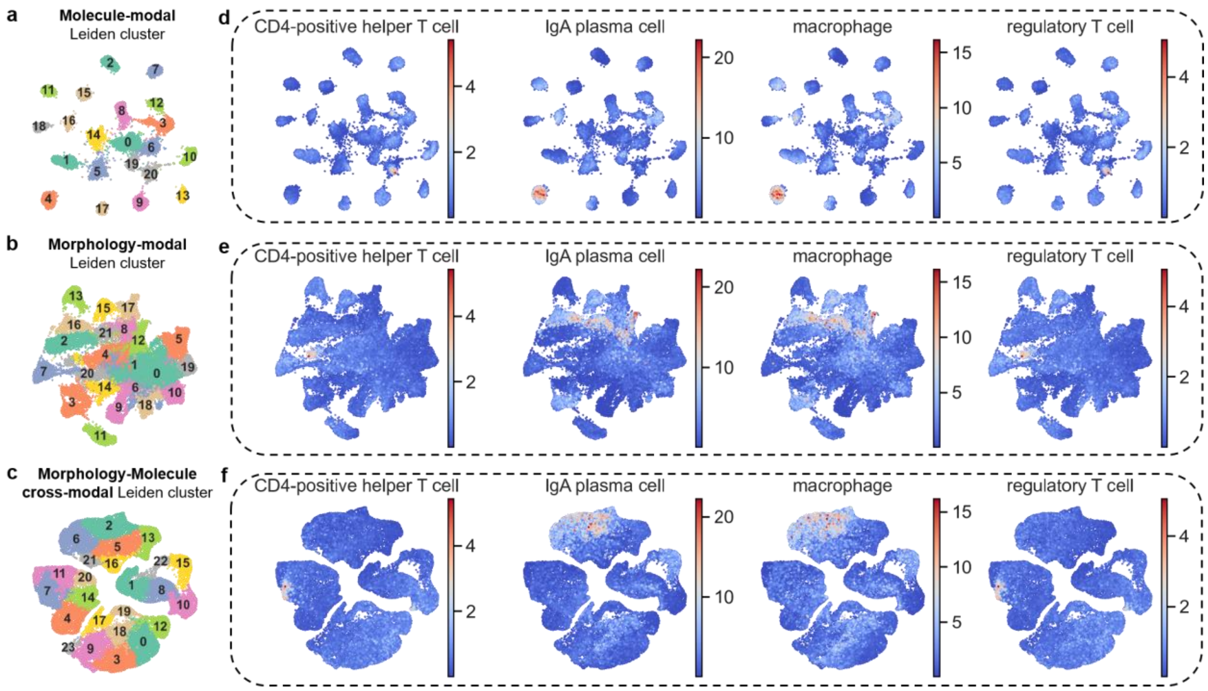

Figure S23. UMAP visualization of Leiden clustering on the embeddings of (a) molecule-only, (b) morphology-only, and (c) morphology-molecule cross-modal data across spots in **stnet** ST samples. (d-f) UMAP appearance of key fine-grained cell types, including IgA plasma cell, proliferating T cell, macrophage cell, and mammary gland epithelial cells, on (d) molecule-only, (e) morphology-only, and (f) morphology-molecule cross-modal embeddings, respectively. The color indicates the abundance values of specific cell types.

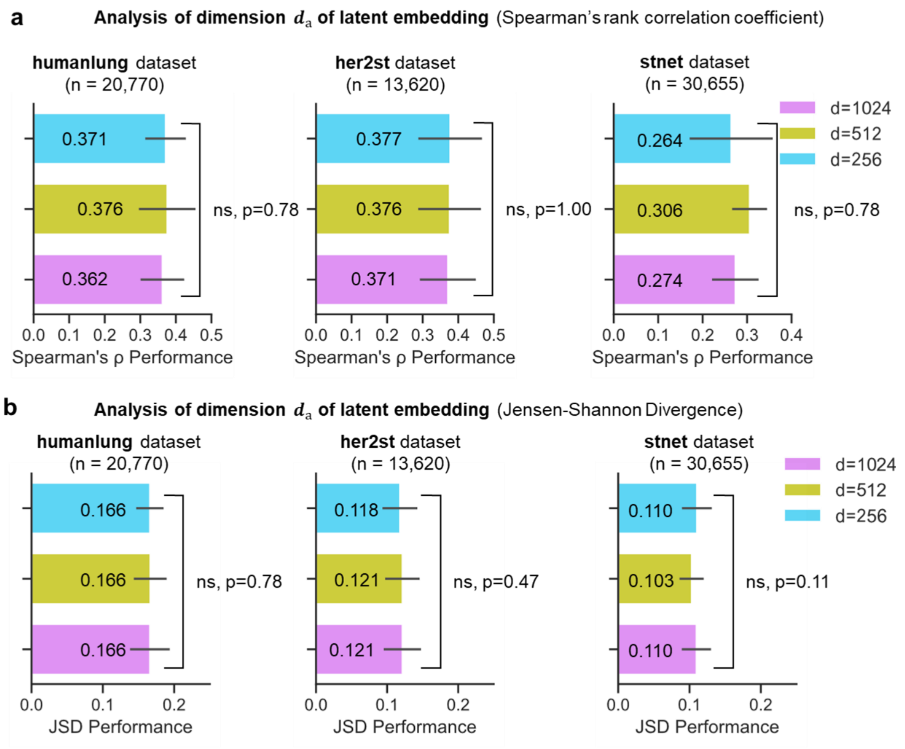

Figure S24. Evaluation results of Ablation study of **Dimension** d_a **of latent embedding** in terms of (a) Spearman's ρ correlation and (b) Jensen-Shannon Divergence (JSD) metrics across three datasets (humanlung, her2st, and stnet). Mean values are shown on the plot with standard deviation indicated by error lines. Group comparisons were analyzed using Friedman test, where 'ns' denotes non-significant differences ($p \geq 0.05$).
